# Mass spectrometry proteomics for studying mitostasis

**DOI:** 10.1002/pro.70673

**Published:** 2026-06-15

**Authors:** Lakshita Sharma, Sristi Chakroborty, Hirak Das, Silke Oeljeklaus, Julian Bender, Bettina Warscheid

**Affiliations:** ^1^ Chair of Biochemistry II, Theodor Boveri‐Institute, Biocenter University of Würzburg Würzburg Germany

**Keywords:** complexome profiling, dynamic SILAC, interactome analysis, mitochondria, nascent proteomics, protein import stress, proteome dynamics, proteostasis, proximity labeling, quantitative mass spectrometry

## Abstract

Maintaining mitochondrial integrity and function is fundamental to cellular homeostasis. Cells rely on coordinated protein quality control (QC) systems—including intricate chaperone‐protease networks, the ubiquitin‐proteasome system, and cytosolic surveillance pathways—that together form a dynamic, cell‐wide mitostasis network governing the import, folding, synthesis, and degradation of mitochondrial proteins. Disruption of mitochondrial homeostasis, for example, by impairing mitochondrial protein import, induces proteotoxic stress and contributes to human disease. Mass spectrometry (MS)‐based proteomics has established itself as an indispensable method to dissect mitostasis at unprecedented depth by enabling systematic quantitative analysis of protein abundance, localization, interactions, stability, and dynamics. In this review, we highlight state‐of‐the‐art MS technologies and multifaceted proteomics approaches used to study mitostasis on a proteome‐wide level. These functional analysis approaches build on quantitative MS methods employing label‐free, metabolic, and chemical labeling strategies, which allow precise tracking of proteome dynamics in response to different cellular conditions including stress. Spatial and interaction‐based approaches, such as affinity purification‐MS, proximity labeling, and complexome profiling, provide detailed insight into the organization and regulation of the complex mitochondrial organizing system, chaperone networks, and protein QC pathways. Furthermore, we discuss advanced methodologies such as nascent chain and dynamic proteomics strategies, which offer a proteome‐wide comprehension of early stress responses and fast regulation. The skillful integration of temporal, spatial subcellular, interaction, nascent, and dynamic proteomics approaches now enables a systems‐level assessment of mitostasis, paving the way for a holistic while nuanced understanding of this essential cellular process and the underlying molecular mechanisms.

## 1 INTRODUCTION

Mitochondria are essential organelles with central roles in cellular energy production, metabolism, biosynthesis of amino acids, lipids and cofactors, redox regulation, and signaling (Chacinska et al., 2009; Pfanner et al., 2019). Maintaining their functional integrity is therefore critical for cellular homeostasis and cell viability. The mitochondrial proteome is highly dynamic and consists of about 900 high‐confidence proteins in yeast (Morgenstern et al., 2017) and over 1100 proteins in humans (Morgenstern et al., 2021; Rath et al., 2021). The vast majority of these proteins are nucleus‐encoded and synthesized as precursors on cytosolic ribosomes. Cytosolic chaperones bind the emerging precursors, maintain them in an unfolded, import‐competent state, and guide them to the mitochondria for import via dedicated translocation machineries in the outer and inner mitochondrial membranes (IM). Most mitochondrial proteins are imported via the translocase of the outer membrane (TOM) complex and transferred to the IM or the matrix via the translocase of the inner membrane (TIM23 complex), which is coupled to the presequence translocase‐associated motor (PAM) for matrix protein import. Multi‐spanning carrier proteins of the IM are inserted via the carrier translocase of the inner membrane (TIM22 complex) (Pfanner et al., 2019). Inside the organelle, mitochondrial proteins need to be correctly folded and protein complexes assembled properly. Mitochondrial functionality therefore relies on the coordinated interplay between cytosolic and mitochondrial protein synthesis, chaperone networks, mitochondrial translocases, and assembly machineries for the biogenesis of mitochondrial protein complexes such as respiratory chain complexes (Kremer & Rehling, 2024; Pfanner et al., 2019; Richter‐Dennerlein et al., 2015; Voos, 2013). Perturbations along this complex, multistep pathway—such as defects in translation, imbalance in chaperone networks, premature precursor folding, dysfunctional protein translocases and assembly machineries, or reduced mitochondrial membrane potential—can compromise protein import and lead to cytosolic accumulation of non‐imported precursors (Coyne et al., 2023; Liu et al., 2019; Pfanner et al., 2025; Wang & Chen, 2015; Wrobel et al., 2015). Since many mitochondrial precursors contain hydrophobic or low‐complexity sequences and require dedicated folding mechanisms (Chacinska et al., 2009; Voos, 2013), their cytosolic accumulation poses a significant risk of cellular proteotoxicity and, eventually, cell death (Liu et al., 2019; Wang & Chen, 2015; Wrobel et al., 2015).

To counteract these challenges, cells have evolved multiple stress response and quality control (QC) pathways that respond to disturbances in mitochondrial protein import and biogenesis. These include cytosolic mechanisms that prevent the (over‐)accumulation of non‐imported mitochondrial precursors as well as specific mitochondrial surveillance systems that relieve translocase clogging and restore import capacity. Collectively, these mechanisms maintain mitochondrial homeostasis, referred to as mitostasis, and thereby also cellular proteostasis. Examples include attenuation of global cytosolic translation to limit new precursor synthesis (Wang & Chen, 2015; Wrobel et al., 2015), activation of a transcriptional program to strengthen the cellular proteostasis network (Boos et al., 2019), elevation of proteasome activity and delivery of non‐imported precursor proteins to the proteasome for their efficient degradation (Kim et al., 2023; Schulte et al., 2023; Wrobel et al., 2015), sequestration of aggregation‐prone precursors into protective compartments (Bertgen et al., 2024; Flohr et al., 2025; Krämer et al., 2023; Ruland et al., 2025), mitochondrial translocation‐associated degradation (mitoTAD) (Mårtensson et al., 2019), the mitochondrial compromised protein import response (mitoCPR) (Weidberg & Amon, 2018), and the mitochondrial unfolded protein response in the matrix (UPRmt) (Münch, 2018; Yoneda et al., 2004; Zhao et al., 2002). For more details about mitostasis pathways, we refer readers to recent reviews (Ahola et al., 2019; Boos et al., 2020; den Brave et al., 2024; Deshwal et al., 2020; Mukhtar et al., 2023; Müller & Hoppe, 2024; Pfanner et al., 2025; Ravanelli et al., 2020).

The dynamic nature of mitostasis, which orchestrates continuous changes in protein synthesis, localization, interactions, stability, assembly/disassembly, and turnover, requires analytical approaches capable of specifically capturing proteome‐wide alterations with high sensitivity and high depth. Modern mass spectrometry (MS)‐based proteomics uniquely enables the quantitative analysis of thousands of proteins in a single experiment, meeting the demands of monitoring proteome‐wide changes in protein abundance, dynamics, stability, and interactomes as well as multiprotein assemblies and complexomes in space and time (Aebersold & Mann, 2016; Christopher et al., 2021; Le Sueur et al., 2022; Liu et al., 2024; Mateus et al., 2020; Schulte et al., 2023; van Bergen et al., 2022; Wu et al., 2024).

In this review article, we cover the key proteomics methods and MS technologies for dissecting the molecular mechanisms underlying mitostasis. The protein‐centric functional approaches we describe are based on high‐resolution quantitative MS in combination with biochemical methods ranging from cell fractionation, organelle purification, separation of native multiprotein assemblies, enrichment of protein complexes, and proximity labeling (PL) to metabolic pulsed and nascent protein labeling and thermal shift assays. We first discuss the basics of bottom‐up proteomics covering sample processing and liquid chromatography‐mass spectrometry (LC–MS) analysis, with a focus on quantitative MS technology. Second, we outline the main functional proteomics approaches applied to identify and characterize mitostasis processes. Lastly, we provide an overview of the application and impact of these multifaceted proteomics methods on the flourishing field of mitostasis research.

## 2 MASS SPECTROMETRY‐BASED PROTEOMICS

MS technologies and, therefore, proteomics approaches using them are multifaceted and, depending on the biological system and research questions to be addressed, different experimental procedures must be followed. Here, we focus on experimental workflows and methods commonly used in MS‐based proteomics, which can be divided into sample processing, peptide sequencing, and quantification technologies.

### 2.1 Sample processing

5Modern MS approaches enable the systematic analysis of proteins in various biological samples of different complexities, ranging from the identification of a purified protein to thousands of proteins in a whole cell lysate. A priori knowledge about the identity of proteins is not required, which is distinctive from the antibody‐based detection of proteins through immunoblot analysis. However, the coupling of biochemical assays to MS technology requires some consideration with respect to sample preparation (Rogers & Bomgarden, 2016). Detergents such as digitonin for membrane solubilization or sodium dodecyl sulfate (SDS) for protein denaturation interfere with MS analysis and must be removed by gel electrophoresis, acetone precipitation, filter‐aided sample preparation (Wiśniewski et al., 2009), or protein aggregation capture methods such as SP3 (Hughes et al., 2019). In a typical bottom‐up proteomics workflow, proteins are reduced and alkylated, enzymatically cleaved to peptides, desalted, and analyzed using ultra‐high performance liquid chromatography (UHPLC) coupled to electrospray ionization (ESI) tandem mass spectrometry (MS/MS). In gel‐based LC–MS analysis, proteins are separated by sodium dodecyl sulfate–polyacrylamide gel electrophoresis (SDS‐PAGE) according to their molecular mass and further processed in the polyacrylamide matrix, whereas in an in‐solution experiment, proteins are processed for LC–MS without additional separation. Most commonly, the endoprotease trypsin is used to enzymatically cleave proteins into peptides with an arginine or lysine residue at the C‐terminus. To increase the number of protein identification (IDs) and their sequence coverage, different proteases (e.g., trypsin, chymotrypsin, LysC, GluC, and AspN) can be used in parallel or in sequence to generate complementary sets of peptides (multi‐protease digestion) (Swaney et al., 2010). In addition, peptide mixtures can be fractionated at high pH using reversed‐phase liquid chromatography which is orthogonal to low‐pH UHPLC coupled to MS. Both strategies have been used to systematically map the yeast and human mitochondrial proteome (Morgenstern et al., 2017, 2021; Sickmann, Mreyen, & Meyer, 2003).

### 2.2 Tandem mass spectrometry

**FIGURE 1:**
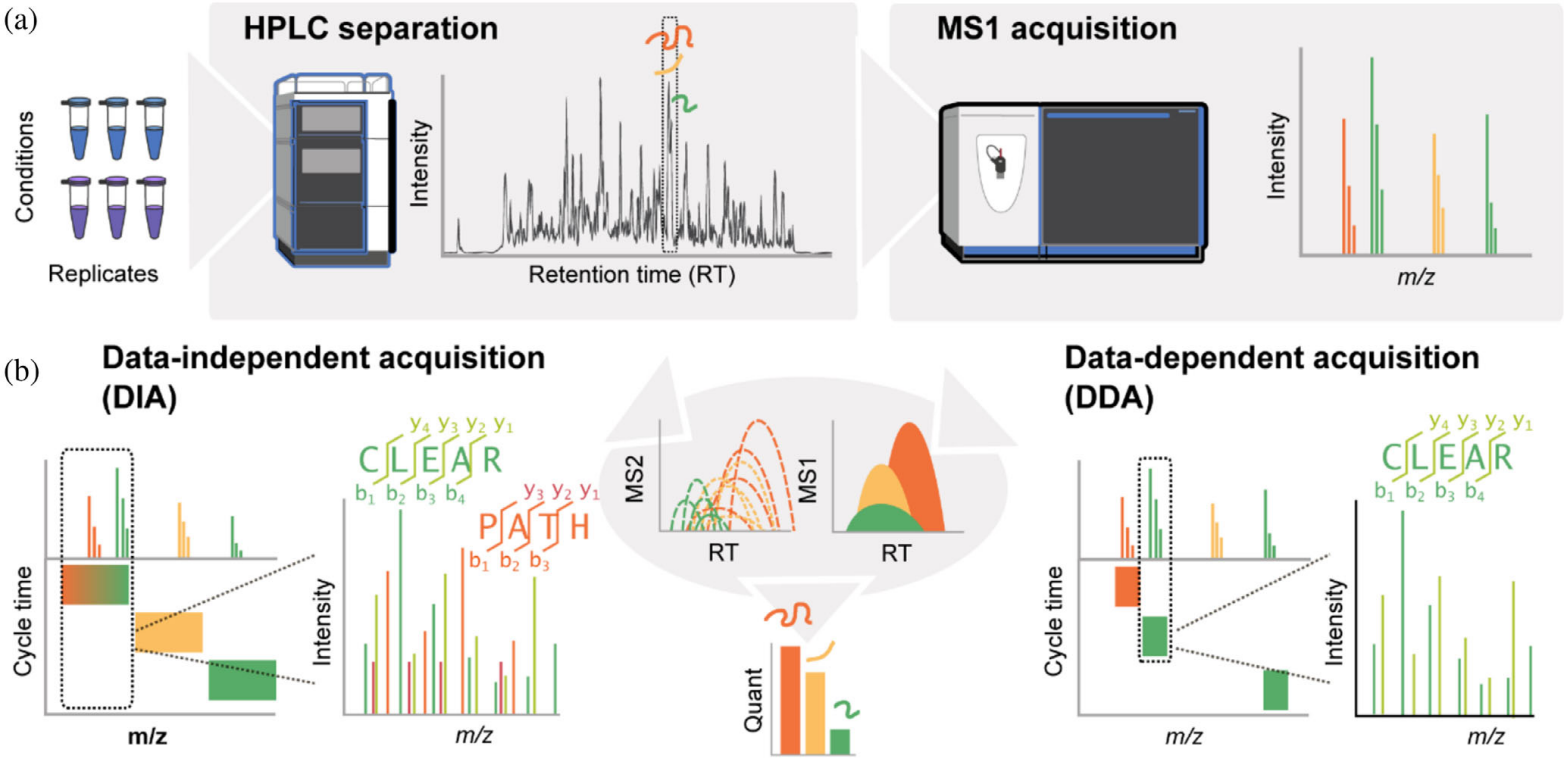
Mass spectrometry (MS) technologies in quantitative proteomics. (a) Peptide mixtures generated from, for example, stressed and non‐stressed (control) cells in biological replicates are separated by their hydrophobicity using reversed‐phase high‐performance liquid chromatography (HPLC) followed by electrospray ionization and injection into a high‐resolution tandem MS system. Peptides of different mass‐to‐charge (*m/z*) ratios eluting from the LC column at the same retention time (orange, yellow, and green) will produce distinct peaks in an MS1 spectrum. (b) Triggering of subsequent MS2 scans depends on the type of MS experiment. In data‐independent analysis (DIA), pre‐defined windows of the MS1 range are selected and fragmented, producing complex mixed MS2 spectra. However, this approach ensures fragmentation of all possible precursor ions independent of their measured intensities. In contrast, data‐dependent acquisition (DDA) selects the N most intense ions on the MS1 level for fragmentation, with *N* typically ranging from 6 to 50. While the likelihood of producing mixed MS2 spectra is reduced compared to DIA, low‐abundant peptide ions might not be selected for fragmentation (see yellow peak). Label‐free peptide quantification is achieved by integrating the peak areas of MS1 (both approaches) or MS2 (most typical for DIA) peaks.

In a typical LC–MS analysis, peptides eluting in an acidic solvent from the LC column are ionized by electrospray and directly transferred to the mass analyzer region of the mass spectrometer. Independent of the MS system used, mass‐to‐charge (*m/z*) ratios and relative ion intensities of protonated peptide ions are recorded in the first stage of the analysis (MS1 scan, Figure 1a). To unequivocally determine the identity of several thousand to hundreds of thousands of peptides in a single analysis, a second stage of analysis is needed, referred to as MS2 scan. In high‐resolution MS1 scans, peptide masses are determined, whereas peptide sequences are deduced from MS2 scans (Steen & Mann, 2004). Together, this type of analysis is termed MS/MS.

In LC–MS/MS, consecutive cycles of MS1 and MS2 scans are performed. After determining accurate *m/z* ratios of all peptides present at a certain time in MS1, precursor peptides are sequentially isolated and fragmented for MS2. Modern MS systems used in proteomics are typically hybrid (or tribrid) instruments with a configuration of two (or three) separate mass analyzers for tandem‐in‐space measurements. Two main principles of peptide precursor selection for untargeted experiments exist (Figure 1b). In the conventional data‐dependent acquisition (DDA) scheme, the most intense N peptide ion species (e.g., TOP10 with *N* = 10) are sequentially selected with narrow isolation windows (±0.5–2 Da, depending on the instrument) for fragmentation by collision‐induced dissociation (CID) or higher‐energy collisional dissociation (HCD) using an inert gas (N_2_). A limitation of DDA is the co‐isolation of different precursor ions resulting in chimeric MS2 spectra (Frejno et al., 2025), which may hamper correct spectrum annotation if chimeric spectra are not expected by the search software. More recently developed data‐independent acquisition (DIA) technology embraces precursor co‐isolation by scanning pre‐defined *m/z* windows in MS2 scans and using specialized software tools to deconvolute the resulting mixed spectra. Advancements in both software and hardware now enable narrow window DIA with scan rates of above 100 Hz (Guzman et al., 2024), combining the advantages of systematic precursor ion selection with MS2 spectra of lower complexity. In both approaches, the acquired fragmentation spectra are searched against a reference sequence database to infer peptide sequences followed by protein assembly using dedicated software for DIA or DDA analysis (see Table 1; Lou & Shui, 2024). High‐end MS‐DIA analysis in conjunction with short LC gradients (10–30 min) yields high proteome coverage (>9000 protein IDs in human cell lines), with low missingness in data and, thus, high data completeness including coverage of low‐abundance protein, which allows for studying mitochondrial stress responses and cellular proteostasis processes on a proteome‐wide scale at unprecedented depth.

**TABLE 1:** Suggested software tools for data analysis.

Software	Application	References
MaxQuant	Database search. Typically used for DDA. DIA‐capable. Free software. Reference software for DDA analysis. Implements TMT correction factors.	Cox and Mann (2008), Tyanova et al. (2016)
Proteome Discoverer	Database search. DIA and DDA. Proprietary software. Programming of analysis workflows using graphical nodes for easy visual inspection. Comes with many analysis workflows for Thermo Scientific MS instruments. Implements TMT correction factors.	Not applicable
Spectronaut	Database search. DIA. Proprietary software. Typically employed for the identification of post‐translational modifications from DIA experiments.	Not applicable
MSFragger	Database search. Typically used for DDA. DIA‐capable Open‐source software. Modern faster alternative to MaxQuant.	Kong et al. (2017)
Fragpipe	Computational platform combining several software tools. DDA and DIA. Open‐Source software. Typically uses MSFragger for DDA and DIA‐NN or MSFragger‐DIA for DIA quantification but also provides downstream tools for post‐processing and validation.	Yu et al. (2023)
DIA‐NN	Database search. DIA. Open‐Source software. Uses deep neural networks for peptide‐centric initial search and spectrum centric signal processing for removing interference in highly complex DIA spectra.	Demichev et al. (2020)
SAINTq	AP‐MS (DDA and DIA). Bayesian modeling of false‐ and true‐positive‐rate of different bait‐prey interactions. Enables assessment of non‐specific interactions using control experiments or implicit. Requires experiments with several bait and control experiments.	Teo et al. (2016)
NOVA	Complexome profiling Class 1. Enables hierarchical clustering with different distance metrics, native mass calibration and various data visualization tools	Giese et al. (2015)
COPAL	Complexome profiling Class 1. Integration of data from multiple experiments.	Van Strien et al. (2019)
ComPrAn	Complexome profiling Class 1. R package for complexome analysis based on two labeled (e.g., SILAC H/L) channels.	Páleníková, Harbour, Ding, et al. (2021)
ComplexFinder	Complexome profiling Class 1. Python‐based pipeline that utilizes machine‐learning to predict participation of a particular protein in multiple protein complexes and identify novel protein–protein interactions	Nolte and Langer (2021)
Gaussian Interaction Profiler	Complexome profiling Class 2. Uses a Gaussian mixture model to represent complex profiles. Assesses the power of different metrics for defining true clusters based on known interacting proteins.	van Strien et al. (2024)
CCProfiler	Complexome profiling Class 2. R package. Open‐source software. Developed for the analysis of DIA (sequential window acquisition of all theoretical mass spectra) data. Implements a complex‐centric analysis in which prior knowledge about protein complexes is used to find corresponding evidence in the MS data.	Bludau et al. (2020)

Abbreviations: AP‐MS, affinity purification combined with MS; DDA, data‐dependent acquisition; DIA, data‐independent acquisition; MS, mass spectrometry; SILAC, stable isotope labeling by amino acids in cell culture; TMT, Tandem Mass Tags.

### 2.3 Quantitative MS techniques

Mitochondrial stress responses are multifaceted, ranging from protein mislocalization, removal of misfolded or aggregated proteins, to changes in transcriptional and translational programs. To uncover these processes, quantitative information about proteins is key (Bantscheff et al., 2012). In quantitative LC–MS analysis, relative changes in protein abundance and dynamics are measured by integrating peptide ion intensities over the retention time of the chromatographic separation (peak areas). Note that ionization efficiencies can differ between peptides, and so do their measured intensities. Consequently, the same peptides in different samples are quantitatively compared. Quantitative MS comes in different flavors, which can be divided into label‐free and stable isotope labeling of either peptides by chemical tagging or proteins during cellular translation.

#### 2.3.1 Label‐free quantification

Label‐free quantification compares peak areas of peptides measured in separate LC–MS runs and is in principle not limited by sample size. Commonly used search engines such as the MaxQuant software suite (Cox & Mann, 2008) perform retention time alignment between LC–MS runs and extract peak areas from high‐resolution MS1 scans, but summed intensities of fragment ions belonging to a common precursor can equally be used as implemented, for example, for the analysis of DIA data in DIA‐NN (Demichev et al., 2020). Depending on whether intensities in a single MS acquisition or between MS acquisitions should be compared, raw signal intensities are commonly modified to increase precision of the comparison. To counteract that protein groups of different lengths and compositions produce a different number of peptides, intensity‐based absolute quantification (iBAQ) (Schwanhäusser et al., 2011), normalizes intensities by the number of its measurable tryptic peptides. Similarly, peptide intensities can be normalized to the summed intensity of histones which allows protein copy number estimation without adding internal peptide or protein standards (Wiśniewski et al., 2014). Label‐free comparison of protein groups between MS acquisitions is typically achieved by establishing a system of pairwise peptide ratios which is then used to normalize the total protein group intensities (MaxLFQ approach) (Cox et al., 2014). While popular and methodologically simple, label‐free MS typically suffers from inter‐run variability introduced through biological or technical variation (e.g., in sample processing or LC performance over time) as well as missing values in sample sets due to stochastic sampling of precursor ions, primarily observed in DDA, or peptide intensities below the detection limit.

#### 2.3.2 Chemical stable isotope labeling

**FIGURE 2:**
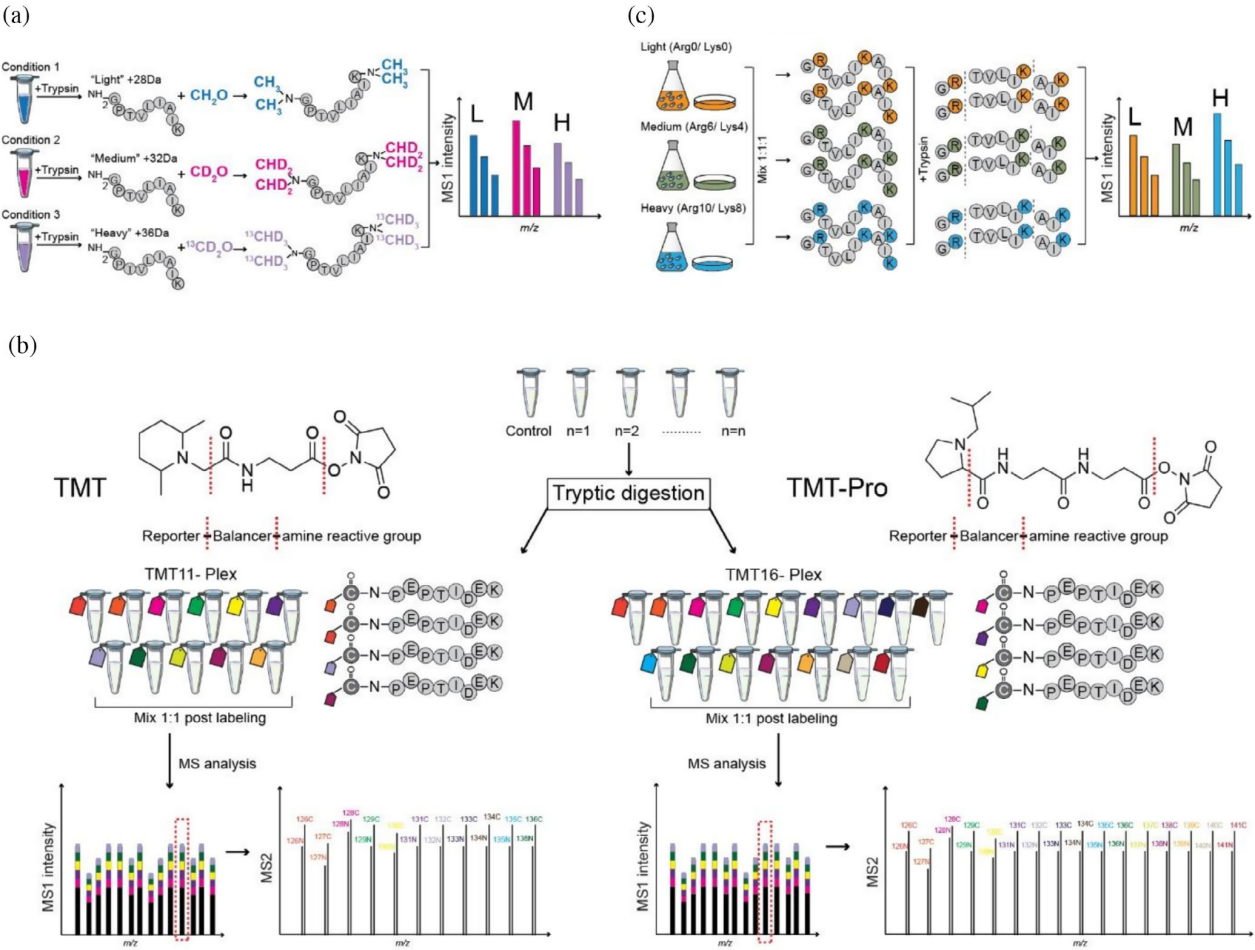
Chemical and metabolic labeling methods. (a) Dimethyl labeling involves the covalent modification of peptide N‐termini and lysine side chains with light (+28 Da), medium (+32 Da), or heavy (+36 Da) dimethyl isotopologues after protein digestion using trypsin. Differentially labeled peptides exhibit distinct mass shifts (e.g., 4 Da) in the MS1 spectrum, enabling relative quantification based on MS1 peak intensities of peptide pairs and triplets. (b) Isobaric labeling. Tandem mass tags (TMT) labeling reagents are covalently attached to primary amines in the peptides via their peptide‐reactive group followed by mixing of differentially labeled peptides in equimolar amounts and LC–MS analysis. The labels exhibit identical nominal masses, resulting in a single, overlapping MS1 peak that facilitates efficient precursor ion selection in data‐dependent acquisition and subsequent peptide identification via MS2 fragmentation. Upon fragmentation, reporter ions are released from the labels, enabling relative quantification based on their intensities in the MS2 spectrum. (c) In stable isotope labeling by amino acids in cell culture (SILAC), unlabeled or isotopically labeled lysine (Lys0, Lys4, or Lys8) and arginine (Arg0, Arg6, or Arg10) are incorporated into newly synthesized proteins during cell growth. After proteolytic digestion and mass spectrometric analysis, the incorporation of heavy isotopes results in characteristic mass shifts detectable in the MS1 spectrum. Quantitative comparison is achieved by evaluating the relative peak areas of the differentially labeled peaks in MS1. MS, mass spectrometry.

Chemical labeling of peptides with stable isotope‐encoded tags allows to mix peptides and analyze them in a single MS run, thereby avoiding inter‐run variability and the associated variance (Figure 2a). In commonly used stable isotope labeling technology, the peptide primary amino groups (i.e., the N‐terminus and the ε‐amino group of lysine) are derivatized with stable isotope labels, whereas reactive thiol groups of cysteine residues are specifically targeted in conjunction with thiol trapping methodology in redox proteomics applications (Brandes et al., 2013; Jonak et al., 2024; Topf et al., 2018). A simple and cost‐efficient proteome‐wide stable isotope labeling approach is dimethyl labeling which uses up to three isotopologues of cyanoborohydride (introducing mass shifts of 28, 32, and 36 Da) for peptide quantification at the MS1 level (Boersema et al., 2009). While this multiplexing technology alleviates inter‐run variance, peptide doublets and triplets detected in MS1 scans result in peak crowding (increasing the chance of random assignments and false positives) with a decrease in the overall sensitivity as the total maximum ion current per scan is fixed (i.e., signal intensities of single peptide peaks are distributed to two or three peaks for all peptides in the analysis). As chemical labeling is a covalent mass modification, all software tools that enable the quantification of post‐translational modifications (see Table 1) can also be applied to chemical labeling.

#### 2.3.3 Isobaric peptide labeling

Isobaric labels such as isobaric tags for relative and absolute quantitation (iTRAQ) (Ross et al., 2004), or tandem mass tags (TMT) (Dayon et al., 2008; McAlister et al., 2012) circumvent the increase in MS1 complexity by shifting all MS1 peaks to a common *m/z* ratio, with the quantitative information from each sample encoded in the intensity of diagnostics fragment ions which can only be detected upon MS2 fragmentation (Figure 2b). For this, the primary amine‐reactive isobaric reagents are composed of different versions of isotope‐labeled reporter and balancer groups, allowing multiplexing experiments. As differentially labeled peptides are isotopomers, they elute at identical retention time and *m/z* ratios in MS1, enabling their co‐isolation and HCD fragmentation to liberate reporter groups with an intensity proportional to the number of labeled peptides before mixing. Peptide ratios are then inferred by comparing intensities of the low *m/z* reporter ions recorded in the MS2 scans. Main advantages of isobaric labeling are higher data completeness and enhanced sensitivity as low‐abundant peptides in one sample will be co‐isolated with their more abundant counterparts in other samples or a dedicated “booster” channel (i.e., a pooled sample from the entire sample set) (Yi et al., 2019). However, accurate peptide quantification based on MS2 reporter ion intensities can be confounded by ratio compression due to co‐isolation of another MS1 envelope which will contribute reporter ion intensities (Ow et al., 2009). To address this, advanced MS3 methodology enabling to select only ions for reporter ion release which belong to the same peptide has been implemented but require specific instrumentation (McAlister et al., 2014) or quantification is based on complementary ions (i.e., the balancer group‐peptide conjugates), which still retain the fragment ion information, but these are more difficult to detect (Johnson et al., 2021).

While iTRAQ comes with up to eight different variants (i.e., channels), TMT has been expanded from six to up to 11 channels, and with the longer TMTpro variant (Thompson et al., 2019) up to 35 channels, facilitating extensive quantitative proteomics studies under multiple experimental conditions. However, care must be taken to ensure sufficient labeling of primary amines but control side reactions toward histidine, serine, threonine, and tyrosine residues (Zecha et al., 2019). Moreover, TMT reagents still contain low amounts of naturally occurring isotopes in the non‐labeled atoms and insufficient enrichment of heavy versions of the labeled atoms contribute satellite peaks which leads to bleeding of intensity across channels (termed crosstalk) during the MS analysis (Brenes et al., 2019). While software can to some degree account for this by taking the specific reagent purities for each TMT batch into account (see Table 1 for a list of software tools), using an experimental design that minimized global interference (Gerault, 2025) is recommended.

#### 2.3.4 Metabolic labeling

Stable isotope labeling by amino acids in cell culture (SILAC) (Ong et al., 2002) can be considered as one of the most robust and accurate techniques in quantitative MS. The method is based on the metabolic incorporation of amino acids—typically arginine and lysine—labeled with non‐radioactive stable isotopes (^2^H, ^13^C, and/or ^15^N) into proteins during cell growth (Figure 2c). This results in a defined mass difference between peptides from labeled and unlabeled cells. For accurate quantification, near‐complete incorporation of the isotope‐coded amino acids into the proteome (>95%) and careful control of the metabolic conversion of heavy arginine to heavy proline (Bendall et al., 2008; Bicho et al., 2010) are required. Since wildtype stains of *Saccharomyces cerevisiae* can synthesize all amino acids, SILAC experiments using yeast are generally performed with strains that are auxotrophic for the amino acids selected for metabolic labeling (Oeljeklaus et al., 2014). Alternatively, the “native SILAC” strategy can be employed, which enables complete labeling of prototrophic yeast strains with stable isotope‐coded arginine and lysine (Dannenmaier et al., 2018; Dannenmaier, Oeljeklaus, & Warscheid, 2021). By using “light,” “medium,” and “heavy” isotopic forms of arginine (Arg0, Arg6, and Arg10) and lysine (Lys0, Lys4, and Lys8), SILAC allows multiplexing of up to three samples.

In a standard SILAC experiment, differentially labeled cell populations from different experimental conditions are typically mixed immediately after harvesting or cell lysis. Similar to chemical peptide labeling, this drastically reduces variance from inter‐run variability but does so even before digestion. Relative differences in protein abundance between samples are determined based on intensities or peak areas of proteolytic SILAC peptide pairs or triplets in MS1 spectra. Commonly used software tools for calculating protein abundance ratios are described in Table 1.

## 3 FUNCTIONAL PROTEOMICS APPROACHES

### 3.1 Charting protein abundance and localization

#### 3.1.1 Whole cell and mitochondrial proteomics

Recent advancements in MS technologies and high‐resolution instrumentation have paved the way for virtually full proteome analysis without the need for fractionation at the cellular (e.g., by differential centrifugation) or protein/peptide level (e.g., using SDS‐PAGE or high pH reversed‐phase fractionation). To date, 8000–10,000 proteins can be readily identified and quantified in whole lysates of mammalian cells in a single analysis employing short LC‐gradients (e.g., 10–30 min) and DIA using high‐end MS systems (e.g., timsTOF Pro or Orbitrap Astral) (Guzman et al., 2024; Skowronek et al., 2022). In conventional mitochondrial proteomics, highly purified mitochondria are analyzed by LC–MS to increase sensitivity and depth (Gaucher et al., 2004; Mootha et al., 2003; Sickmann, Reinders, et al., 2003). Subtractive proteomics of crude versus pure mitochondrial fractions allowed establishing the mitochondrial compendium, MitoCarta (Pagliarini et al., 2008; Rath et al., 2021), as well as high‐confidence mitochondrial proteomes of yeast and human cells (Morgenstern et al., 2017, 2021). Using quantitative MS technology (e.g., label‐free or SILAC) proteins of mitochondrial residency and co‐purified contaminants are distinguished based on their enrichment or depletion in gradient‐purified mitochondria compared to crude mitochondrial fractions. However, the analysis of isolated mitochondria largely precludes insight into protein alterations outside the organelle, which is in particular relevant for identifying cellular proteostasis pathways activated by mitochondrial dysfunction.

#### 3.1.2 Spatial proteomics

Investigating proteins in a cellular context (i.e., in whole cell lysates, soluble and insoluble fractions, or crude mitochondrial fractions) enables to trace mislocalized proteins during mitochondrial import stress and to identify proteostasis factors and pathways activated in response to mitochondrial dysfunction. In addition, many mitochondrial proteins are multi‐localized, that is, they can adopt context‐specific roles across compartments through relocalization (Yogev & Pines, 2011). Spatial subcellular proteomics technology establishes cellular proteome maps with high subcellular resolution allowing to follow proteome remodeling during stress and disease conditions (Breckels et al., 2024). To uncover the subcellular location and abundance of proteins (as well as their isoforms) on a proteome‐wide level, biochemical fractionation, immunoprecipitation, PL or a combination of them has been employed (reviewed in Christopher et al., 2021). Spatial proteomics approaches using differential centrifugation and/or density‐gradient centrifugation takes advantage of de Duve's principle, which states that proteins with similar distribution patterns across subcellular fractions likely reside in the same organelle (de Duve et al., 1955), and has been used to profile the abundance distributions of thousands of proteins by MS and correlate them to bona‐fide subcellular marker proteins, a method termed protein correlation profiling (PCP). Typically, advanced computational analyses such as machine learning and clustering approaches are required to assign proteins to each subcellular niche. For example, hyperLOPIT and variants have been used to create detailed spatial maps of cells, as well as to study proteome dynamics (Christopher et al., 2021; Mulvey et al., 2017, 2021).

### 3.2 Interrogating multiprotein assemblies

#### 3.2.1 Affinity purification and proximity labeling

**FIGURE 3:**
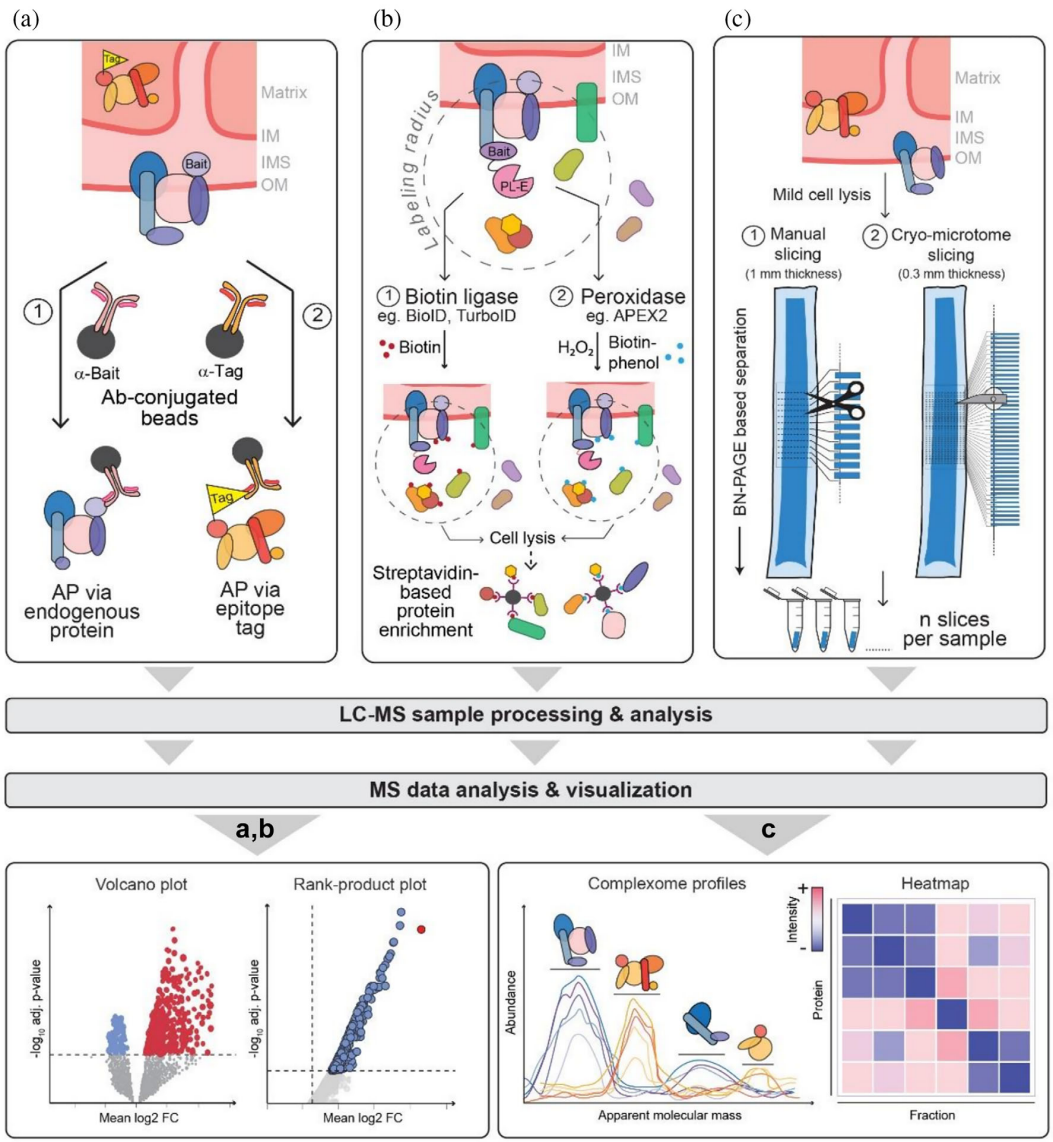
Mass spectrometry (MS)‐based proteomics strategies for the analysis of protein assemblies. (a) In classical affinity purification (AP)‐MS experiments, protein complexes are enriched from whole cell lysates or organelle‐enriched fractions using antibodies (Ab) specifically recognizing the bait protein or via epitope tags fused to the bait. Following LC–MS analysis and MS data processing, statistical significance is assessed using, for example, the limma or rank‐product test, and the resulting data are visualized as volcano or rank‐product plot, in which the mean of the log‐transformed bait‐versus‐control protein ratios (log fold‐change) is plotted against the negative log_10_ of the corresponding adjusted (adj.) *p*‐value. (b) Proximity labeling (PL) approaches rely on the biotinylation of proteins in close proximity to the bait which is fused to a proximity‐labeling enzyme (PL‐E). The PL‐E can be a biotin ligase with a labeling radius of ~10 nm (BioID and related enzymes) or a peroxidase with ~20 nm radius (APEX/APEX2). Protein biotinylation requires the addition of biotin or biotin‐phenol and H_2_O_2_, respectively. Following cell lysis under harsh conditions, biotinylated proteins are enriched using streptavidin and analyzed by LC–MS. Data analysis and visualization are performed as described in (a). (c) Complexome profiling, used for the interrogation of large protein assemblies under native conditions, requires mild lysis and solubilization conditions (e.g., using digitonin) to preserve physiological (sub)complexes of soluble and membrane‐bound assemblies. Protein complexes are generally separated by blue native (BN)‐PAGE, followed by cutting the gel manually into 60–116 slices or into >200 slices using cryo‐slicing, and LC–MS analysis. The resulting MS intensities per fraction are used to establish protein abundance profiles. Assignment of proteins to distinct protein complexes is based on similarities between abundance profiles. Data are often subjected to hierarchical clustering and visualized as heat maps. IM/OM, inner/outer mitochondrial membrane; IMS, intermembrane space.

The study of multiprotein complexes and larger protein interactomes using protein affinity purification combined with quantitative MS (qAP‐MS) (Gingras et al., 2007; Liu et al., 2024; Oeljeklaus et al., 2009; Wu et al., 2024) has significantly shaped our current understanding of how mitochondrial protein machineries are involved in various processes ranging from mitochondrial protein import processes, inner membrane organization and cristae formation, mitochondrial fission and fusion to bioenergetics. Traditionally, epitope tags or specific antibodies are used to selectively enrich a protein of interest (the “bait”) together with associated proteins (Figure 3a). While mitochondrial protein complexes have typically been purified from crude mitochondrial fractions to increase specificity and sensitivity, whole cell extracts can be used as well, particularly when studying stress response or QC pathways at the mitochondrial surface. In either case, cell lysis and solubilization of mitochondrial membrane proteins should be performed under mild conditions to maintain the integrity of multiprotein assemblies.

Application of quantitative MS techniques and adequate statistical control of variance enables the identification of true interactors against a complex background of hundreds to thousands of non‐specifically co‐purified background proteins. This has led to the discovery of so far unrecognized components and factors associated with mitochondrial protein import complexes (Gebert et al., 2011, 2012; Opaliński et al., 2018; Özdemir et al., 2025), novel complexes such as the Mitochondrial Contact Site and Cristae Organizing System (MICOS) complex (von der Malsburg et al., 2011) and cooperation between different mitochondrial translocases (e.g., TOM‐SAM) (Qiu et al., 2013) or so far unknown Complex IV assembly intermediates connecting mitochondrial precursor import with respiratory chain assembly and mitochondrial translation regulation (Mick et al., 2012). Combined with chemical crosslinking, qAP‐MS can provide low‐resolution structural information about protein complexes, as shown for the interface of the TOM‐TIM23 supercomplex in the intermembrane space (IMS) of yeast (Gomkale et al., 2021) and the architecture of the human TIM22 complex (Valpadashi et al., 2021). Moreover, cross‐linking of purified mitochondria without enrichment of protein complexes was also used to generate a comprehensive picture of the network of protein structures within the organelle (Linden et al., 2020; Liu et al., 2018).

PL‐based proteomics has evolved as a powerful alternative to qAP‐MS that provides complementary data. It allows mapping of local protein environments and interaction networks in living cells under in vivo conditions (Gingras et al., 2019; Liu et al., 2024; Qin et al., 2021; Samavarchi‐Tehrani et al., 2020) (Figure 3b). In PL approaches, a labeling enzyme is fused to a bait protein or targeted to a specific compartment where it tags proteins with reactive side chains in proximity with covalent modifications which preserve the contact information after protein digestion. Of note, using separate N‐ and C‐terminal fusions of the bait with the labeling enzyme may lead to a more complete proximitome since proteins close to the non‐labeled terminus may be outside the labeling radius and are therefore not detected. Furthermore, when applied to membrane proteins, tagging both termini may provide information about the membrane topology of the protein (Lee et al., 2016) since proximitomes will differ if tags are located on different sides of the membrane. However, the addition of the labeling enzyme may compromise the function or correct subcellular localization of the bait protein. Thus, functionality and proper localization of the fusion proteins should be tested prior to the experiment.

The most frequently used labeling enzymes are variants of mutated bacterial biotin ligases (BioID, BioID2, TurboID, and miniTurbo) (Branon et al., 2018; Kim et al., 2016; Roux et al., 2012), or an engineered ascorbate peroxidase (APEX and APEX2) (Lam et al., 2014; Rhee et al., 2013). Upon addition of biotin, biotin ligase‐derived enzymes convert adenosine triphosphate (ATP) to reactive biotin‐adenosine monophosphate (Roux et al., 2012), which covalently biotinylates lysine residues on nearby proteins within a radius of ~10 nm (Kim et al., 2014). PL using APEX/APEX2 requires the addition of biotin‐phenol and H_2_O_2_. The peroxidase catalyzes the generation of phenoxyl‐radicals, which covalently react with tyrosine (primarily) and other electron‐rich amino acid residues. These phenoxyl‐radicals are very short‐lived (<1 ms) and are considered to enable labeling within a radius of ~20 nm under short labeling conditions (Rhee et al., 2013). However, APEX‐mediated biotinylation can be detected over a broader spatial range under conditions (e.g., prolonged labeling times) when the radicals can diffuse more freely (Hung et al., 2014). Although APEX/APEX2 is used for identifying protein interaction partners, it is particularly powerful for mapping subcellular compartments, as demonstrated in initial APEX studies targeting the mitochondrial matrix and IMS (Hung et al., 2014; Rhee et al., 2013). Depending on the enzyme chosen, labeling times range between 18 h for first generation BioID (Qin et al., 2021) and 10 min for TurboID and miniTurbo (Qin et al., 2021), thereby greatly improving temporal resolution and reducing non‐specific background. APEX2 provides even faster labeling kinetics of seconds up to ~1 min (Qin et al., 2021). Biotinylated proteins or peptides (following proteolytic digest) are then enriched from cell lysates using streptavidin affinity purification and identified by LC–MS.

Since biotin is covalently attached to target proteins during labeling, preservation of native protein–protein interactions—whether stable, weak, or transient—is not required during sample preparation. Consequently, cells can be lysed under harsh conditions (e.g., using SDS or urea), enabling efficient solubilization of membrane‐associated and otherwise poorly soluble proteins. Furthermore, the high affinity of the biotin‐streptavidin interaction permits stringent washing conditions to reduce non‐specific background binding while retaining biotinylated proteins.

It has to be noted that the outcome of qAP‐MS and PL experiments critically depends on the inclusion of suitable controls to differentiate between true interaction partners and non‐specific background binders. Controls for qAP‐MS studies are, for example, cells expressing the non‐tagged version of the bait or the use of non‐specific antibodies. Common controls in PL studies include cells lacking the PL enzyme, omission of the substrate (biotin or biotin‐phenol/H_2_O_2_), expression of an untagged or green fluorescent protein (GFP)‐tagged PL enzyme, or the PL enzyme fused to an unrelated protein with comparable expression levels and similar cellular environment (soluble/membrane‐bound) as the bait (Gingras et al., 2019; Jiang et al., 2025).

The application of any of the quantitative MS techniques described above enables calculation of protein enrichment factors. True components of protein assemblies are characterized by high bait‐to‐control ratios, whereas non‐specific binders show no enrichment. While in AP‐MS experiments, enriched proteins are generally considered to be associated with the bait complex, in PL experiments, this may simply reflect spatial proximity.

To obtain statistically sound and relevant data, experiments must be performed in replicates (*n* ≥ 4). Statistical analysis, in the simplest form, consists of taking the bait‐to‐control ratios and subjecting them to any test for statistical equivalence such as the *t*‐test or using linear regression, for example, in form of the limma package (Smyth, 2005). Both tests assume normality of the underlaying data, which may not be true for highly skewed ratio data from AP‐MS experiments. Instead, non‐parametric tests such as the rank‐product test (Del Carratore et al., 2017) can be used. Another caveat of selective protein enrichment is the high likelihood of missing the corresponding protein in the control condition. For calculating ratios, this requires assuming (termed imputing) a low‐intensity value for these peptides in the absence of a better estimate. In such cases, the rank‐product test can also be applied directly to the intensities from the bait condition, omitting the control altogether. When a sufficiently large number of baits is tested, the non‐specific interactions can be assumed to be prey‐specific and therefore can be estimated from the prey intensities in different controls. For such larger interaction studies, sophisticated tools such as SAINTq (Teo et al., 2016), which explicitly model the probability of each bait‐prey interaction being a false positive should be applied. To present such interaction data, volcano or rank‐product plots, which show the average bait‐control ratio versus the negative log of the multiple testing‐corrected *p* value, are typically used. More reliable interactors have a high bait‐versus‐control ratio and a low *p*‐value (i.e., high −log_10_ *p*‐value) (see Figure 3).

Public databases and resources for protein interaction data are listed in Table 2.

**TABLE 2:** Databases and resources for protein interaction data.

Database/resource	Description	References
Interactome/protein–protein interaction (PPI) databases		
BioPlex 3.0	Large‐scale human interactome from high‐throughput AP‐MS in HEK293T and HCT116 cells. Comprises >100,000 interactions among >13,000 proteins. Reveals cell‐type‐specific remodeling of the interactome.	Huttlin et al. (2021)
BioGRID	Comprehensive interaction repository curating genetic and physical interactions, chemical associations, and post‐translational modifications from >87,000 publications.	Oughtred et al. (2021)
IntAct	Curated molecular interaction database at EMBL‐EBI providing >1 million binary interactions.	Del Toro et al. (2022)
STRING	Database of known and predicted protein–protein interactions from direct physical and functional associations.	Szklarczyk et al. (2023)
Proximity labeling (proximitome) MS studies		
High‐density human mitochondrial proximity interaction network	BioID‐based high‐resolution human mitochondrial protein proximity map with 100 mitochondrial baits from all mitochondrial sub‐compartments. Comprises of 1465 proteins, producing 15,626 unique high‐confidence proximity interactions.	Antonicka et al. (2020)
Proximity‐dependent biotinylation map of a human cell	BioID‐based map of a human cell on the basis of 192 subcellular markers.	Go et al. (2021)
Complexome profiling resources		
MitCOM	High‐resolution mitochondrial complexome of yeast. Covers >90% of the yeast mitochondrial proteome using cryo‐slicing blue native‐MS (csBN‐MS).	Schulte et al. (2023)
Complex Portal	Manually curated, encyclopedic resource of macromolecular complexes from multiple species maintained at European Molecular Biology Laboratory‐European Bioinformatics Institute. Provides stoichiometry, topology, and functional annotations for stable protein complexes.	Meldal et al. (2019)
hu.MAP 3.0	Comprehensive human protein complex map integrating >25,000 MS experiments (AP‐MS, cofractionation‐mass spectrometry, proximity labeling) using machine learning. Identifies >15,000 protein complexes covering ~70% of the human proteome.	Fischer et al. (2025)
CORUM 5.0	Manually curated repository of experimentally characterized mammalian protein complexes (>5200 complexes).	Tsitsiridis et al. (2023)
CEDAR	ComplexomE profiling DAta Resource; open access database for deposition and direct and interactive exploration of CP data	van Strien et al. (2021)

Abbreviation: AP‐MS, affinity purification combined with MS; CP, complexome profiling; MS, mass spectrometry.

#### 3.2.2 Complexome profiling

Interrogation of multiprotein complexes, supercomplexes and protein assembly networks requires preserving their physiologically relevant contacts during analysis. Techniques most often used for separating and profiling native multiprotein complexes are size‐exclusion chromatography (SEC) (Connelly et al., 2018) and blue native (BN)‐PAGE (Wessels et al., 2009). In complexome profiling (CP), native SEC or blue native‐polyacrylamide gel electrophoresis (BN‐PAGE) analysis is coupled with quantitative MS (Cabrera‐Orefice et al., 2021) (Figure 3c) to globally map native protein complexes, the latter approach being preferred for the analysis of mitochondrial assemblies (Fernández‐Vizarra et al., 2021; Guerrero‐Castillo et al., 2017; Guerrero‐Castillo, Krisp, et al., 2021; Guerrero‐Castillo, van Strien, et al., 2021; Heide et al., 2012; Morgenstern et al., 2017, 2021; Páleníková, Harbour, Prodi, et al., 2021; Rawat et al., 2020; Schulte et al., 2023; Stenton et al., 2021; Szczepanowska et al., 2020). To maintain non‐covalent interactions, mild, non‐ionic detergents (e.g., digitonin, dodecyl‐β‐d‐maltoside [DDM], triton X‐100) are used for solubilization of membranes or isolated membrane‐enclosed compartments such as mitochondria before BN‐PAGE separation (Wittig et al., 2006). Since digitonin preserves labile and/or higher‐order membrane protein complexes (e.g., OXPHOS complexes) better than DDM (Schägger & Pfeiffer, 2000; Zheng et al., 2023), it is typically used for investigating mitochondrial membrane protein assemblies (Wittig et al., 2006).

In BN‐PAGE, binding of the anionic dye Coomassie Brilliant Blue G‐250 to proteins and protein complexes camouflages their charge at neutral pH and provides a net negative charge which allows migration toward the anode and separation by size during BN‐PAGE separation (Rapaport, 2013). Standard BN‐PAGE resolves protein complexes ranging from several kilodaltons up to nearly 10 MDa, while large‐pore BN‐PAGE (lp‐BN‐PAGE) has been introduced to improve separation of high‐molecular weight complexes exceeding this mass range (Strecker et al., 2010). Downstream processing of BN gels comprises fixation and staining of proteins followed by horizontal sectioning into discrete fractions. The number of slices determines the resolution of the resulting complexome profile, with early studies typically employing ~24 fractions and more recent approaches increasing this number to 60–116 fractions by manual slicing (Heide et al., 2012; Morgenstern et al., 2021; Patron et al., 2022; Senkler et al., 2017; Wessels et al., 2009). To further enhance resolution and minimize technical variability introduced by manual gel excision, cryo‐slicing BN‐PAGE‐MS (csBN‐MS) was developed (Müller et al., 2016). This strategy increases fractionation depth to more than 200 slices per gel lane. Conventional gel‐based sample processing is then used to generate peptide samples.

After protein digestion and LC–MS/MS analysis, protein abundance profiles are constructed. For this, usually either MaxLFQ or iBAQ values are plotted against the corresponding fraction number. The key advantage of iBAQ is its ability to enable a direct assessment of complex stoichiometries within protein assemblies, thereby facilitating the interpretation of relative subunit composition in multiprotein complexes (Guerrero‐Castillo et al., 2017; Rawat et al., 2020). In the actual CP step, similarities between profiles of different protein groups (and therefore putative native protein complexes) are inferred, either using the complete intensity profile (Class 1 in Table 1) (Giese et al., 2015; Hu et al., 2019; Nolte & Langer, 2021; Páleníková, Harbour, Ding, et al., 2021; Stacey et al., 2017) or by focusing on a subset of individual peaks for clustering (Class 2 in Table 1) (Gorka et al., 2019; Schulte et al., 2023). For Class 1 approaches, metrics such as the Pearson correlation between profiles or the number of shared peaks are usually used for, for example, hierarchical clustering. In some cases, multiple metrics or external information are integrated using machine learning prior to clustering (Hu et al., 2019; Nolte & Langer, 2021; Stacey et al., 2017). In Class 2 approaches, individual peaks are extracted using peak deconvolution tools; their properties are compared, thereby enabling the complex assignment of proteins that take part in more than one complex. ComplexomE profiling DAta Resource (CEDAR, www3.cmbi.umcn.nl/cedar/) was established in 2021 to collect and report the generated CP data from various sources (van Strien et al., 2021).

CP of isolated mitochondria has been extensively used to study OXPHOS complexes and their assembly pathways. For example, BN‐PAGE‐based separation of assembly intermediates allowed clustering of associated proteins based on their quantitative co‐migration profiles. Studies such as Heide et al. (2012) and Guerrero‐Castillo et al. (2017) provide detailed insight into the assembly states of human mitochondrial Complex I and in the process identified several previously unidentified OXPHOS assembly factors, highlighting the role of CP in elucidating assembly pathways of multiprotein assemblies. CP has also been used to study OXPHOS under stress conditions, for example, to reveal remodeling of the OXPHOS machinery under mitochondrial deoxyribonucleic acid ablation and proteostatic stress (Guerrero‐Castillo, van Strien, et al., 2021; Rawat et al., 2020). Additionally, it has been used in combination with pulsed SILAC labeling to characterize Complex I maintenance and repair (Stenton et al., 2021; Szczepanowska et al., 2020).

To reduce measurement time and increase throughput, CP can also be multiplexed using SILAC and TMT labeling (Fernández‐Vizarra et al., 2021; Guerrero‐Castillo, Krisp, et al., 2021; Páleníková, Harbour, Prodi, et al., 2021). However, CP also suffers from the inability to capture highly labile interactions such as the interaction of TOMM70 with the TOM complex, which has been shown to dissociate during analysis (Schulte et al., 2023). Further, despite CP's considerable potential for discovering new components of known multiprotein assemblies or even previously unknown native protein complexes, the association is inferred from co‐migration patterns alone and in many cases, data interpretation is challenging. Thus, validation using orthogonal methods such as AP‐MS is necessary.

Public databases and resources for complexome data are listed in Table 2.

### 3.3 Monitoring proteome dynamics

In conventional MS‐based quantitative proteomics, changes in steady‐state levels of proteins are compared between conditions. However, the cellular proteome is highly dynamic, with protein synthesis and degradation precisely adapting to changing conditions. Thus, to understand cellular proteostasis processes that maintain and restore proteome function, proteome‐wide knowledge about protein synthesis and degradation rates is pivotal. This kinetic dimension is captured through pulse metabolic labeling strategies that distinguish newly synthesized proteins from the pre‐existing pool during subsequent quantitative MS analysis.

#### 3.3.1 Dynamic SILAC

**FIGURE 4:**
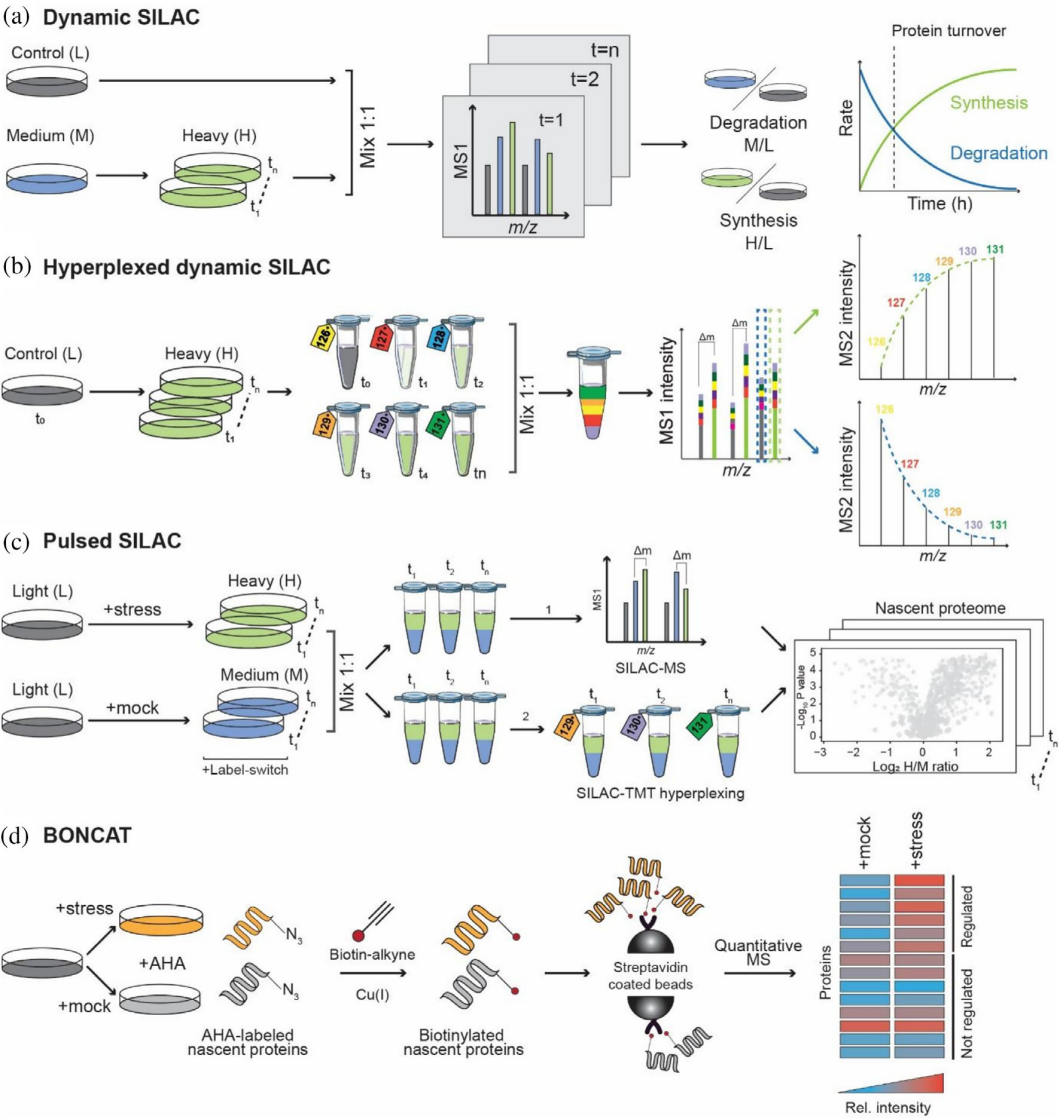
Proteomic strategies to monitor protein turnover and nascent proteins. (a, b) Protein turnover analysis using dynamic stable isotope labeling by amino acids in cell culture (SILAC) methods. (a) Cells initially grown in light (L) medium are shifted to medium‐heavy (M) or heavy (H) SILAC amino acid‐containing medium. Samples collected at multiple time points (*t*_1_–*t*_n_) are mixed 1:1 with an independent control (L) and analyzed by LC–MS. The incorporation of heavy Arg/Lys into newly synthesized proteins and the decrease of medium‐heavy labeled peptides allow quantification of synthesis (H/L) and degradation (M/L) rates from MS1 spectra and thereby proteome‐wide estimation of protein turnover rates. (b) Multiplexed dynamic SILAC approach. Cells are switched from light (time point zero, *t*_0_) to heavy SILAC medium and harvested at successive time points (*t*_1_–*t*_*n*_). Protein samples are digested using trypsin, peptides labeled with tandem mass tags (TMT) and combined in equal ratios for hyperplexed mass spectrometry (MS) analysis. The time‐dimension is encoded in the TMT labels, while pre‐existing and newly synthesized proteins are differentiated by the SILAC labels. Time‐resolved quantification of either degradation (selection of light label in MS1) or synthesis (heavy label in MS1) is achieved through reporter ion signals on the MS2 level. Protein synthesis and degradation curves are calculated for protein turnover estimation as indicated in (a). (c) Pulsed SILAC for nascent chain proteomics. Control and stressed cells are switched from light (L) to heavy (H) or medium‐heavy (M) SILAC medium, respectively. Samples collected at different time points are mixed in equal ratio and sequentially analyzed by LC–MS. Quantification of changes in the nascent proteome is based on M to H peptide peaks in MS1 spectra (1). Alternatively, mixed SILAC samples of different time points can be labeled with TMT, pooled, and analyzed in a single MS experiment (2). Quantification is achieved on the MS2 level for nascent proteins from stressed and control cells encoded by heavy and medium‐heavy MS1 peaks, respectively. (d) Bioorthogonal non‐canonical amino acid tagging (BONCAT) using L‐azidohomoalanine (AHA) for nascent chain proteomics. Cells are incubated with AHA, which is incorporated into newly synthesized proteins under mock or stress conditions. AHA‐containing proteins are selectively conjugated to biotin‐alkyne via Cu(I)‐catalyzed click chemistry, enriched using streptavidin beads, and analyzed by quantitative MS. Relative quantification enables determining changes in the levels of newly synthesized proteins between conditions.

To follow the synthesis and degradation of proteins, SILAC is commonly used for whole‐proteome metabolic pulse labeling. Cells are typically shifted from light to heavy SILAC medium, and the incorporation of the heavy labels is followed over time (Figure 4a,b). Dynamic SILAC was first introduced by Beynon and coworkers (Doherty et al., 2009) to globally determine protein turnover rates in human cells and further refined by Lamond and colleagues (Boisvert et al., 2012) to simultaneously measure protein synthesis, degradation, and turnover in parallel by introducing a “control” channel employing triple SILAC approach (Figure 4a). Notably, global protein and messenger ribonucleic acid (mRNA) turnover analyses showed that protein and mRNAs half‐lives do not correlate (Schwanhäusser et al., 2011), underscoring the need to study dynamics at the proteome level. To combine temporal precision with high‐throughput capability, dynamic SILAC has been coupled to TMT (termed hyperplexing) (Zecha et al., 2018). Relative isotope abundance values, that is, the ratio of one isotope signal to the total peptide signal (e.g., H/[H + L]) across the multiplexed channels, are fitted to first‐order kinetic models to calculate degradation and synthesis rates. In an alternative approach, cycloheximide (CHX) chase assays were coupled to TMTpro multiplexing MS to capture fast proteome dynamics, establishing a proteome‐wide map of ~1000 proteins short‐lived proteins (*t*_1/2_ < 8 h) in human cells (Li et al., 2021). However, in contrast to dynamic SILAC, such pulse‐chase assays require the use of translational inhibitors, inducing global proteostatic imbalance and, therefore, might cause cellular stress.

While dynamic SILAC is typically employed in cell culture systems, it has also been implemented in mouse models to determine protein half‐lives (Ariosa‐Morejon et al., 2021; Claydon et al., 2012; Fornasiero et al., 2018). Estimation of protein turnover in mouse (Fornasiero et al., 2018) showed that protein stability is highly tissue‐specific, whereas mitochondrial proteins displayed similar turnover rates across tissues, including brain, heart, and skeletal muscle, and were generally more stable than non‐mitochondrial proteins.

#### 3.3.2 Nascent proteomics

Capturing changes in the abundance of newly synthesized proteins during stress or during stress recovery requires specific labeling techniques of nascent chains including (i) SILAC amino acids (Figure 4c), (ii) methionine analogs (Figure 4d), or (iii) the aminonucleoside antibiotic puromycin which causes premature termination of translation (reviewed in van Bergen et al., 2022). In the following, we focus on the first two more widely used approaches.

The simplest approach for measuring newly synthesized proteins is pulsed SILAC (pSILAC). Cells are grown in medium containing light (L) versions of the SILAC amino acids arginine (Arg0) and lysine (Lys0) and then shifted to medium containing the heavy (H) versions (e.g., Arg10 and Lys8). By analyzing the incorporation of the heavy Arg/Lys versions into the proteome over time (e.g., 1–24 h) using MS, the fractional synthesis rate of thousands of proteins can be determined based on the calculated H/L ratios (Boisvert et al., 2012; Jovanovic et al., 2015; Schwanhäusser et al., 2011). To directly determine translational responses to stress or upon stress relief, a triple SILAC approach is typically employed enabling to compare newly synthesized proteins between two conditions in the same MS run (Dannenmaier, Desroches Altamirano, et al., 2021; Fierro‐Monti et al., 2013; Hosp et al., 2015; Imami et al., 2021, 2023). In this setup, control and stressed cells are shifted from light to medium‐heavy (M) or heavy SILAC medium, respectively, followed by mixing of cells for joint MS analysis. Changes in the nascent proteome are determined based on M/H peptide ratios, while the pre‐existing protein population (L) is neglected in the analysis.

Although technically straightforward, a limitation of pSILAC is the low abundance of nascent peptides during short labeling intervals, which strongly decreases their detection frequency against a high background of pre‐existing proteins present at early time points. This constraint has been elevated by pSILAC‐TMT hyperplexing with the incorporation of a “booster channel” (also referred to as a material‐rich reference channel). This approach improves quantification and carrier‐assisted identification in multiplexed proteomics as the extra channel with a higher protein amount serves as a calibrator and signal enhancer (Ctortecka et al., 2022; Specht & Slavov, 2021; Yi et al., 2019). The application of this principle to nascent proteomics was shown with the multiplexed enhanced PROtein Dynamics (mePROD) method (Klann et al., 2020). To increase the sensitivity for newly synthesized proteins, the booster channel consists of a fully heavy SILAC‐labeled proteome, which carries sufficient peptide intensities for identifying and quantifying low‐abundance nascent chains after short labeling times. Using mePROD, alterations in the translational program following inhibition of the integrated stress response (ISR) have been quantified to a depth of ~5200 nascent proteins after 2 h labeling time (Klann et al., 2020). The approach enables hyperplexing of multiple time points or conditions in a single run but requires specialized MS technology (e.g., synchronous precursor selection, SPS‐MS3, on tribrid instruments to minimize ratio compression) (Hogrebe et al., 2018), expensive labeling reagents and complex data processing pipelines.

To specifically monitor immediate early translational responses at high sensitivity, advanced nascent proteomics methods have been developed that allow for the specific labeling and enrichment of the nascent proteome pool before MS analysis. This is achieved via the metabolic incorporation of the methionine analog L‐azidohomoalanine (AHA) or homoproargylglycine (HPG) (Dieterich et al., 2006; Hatzenpichler et al., 2014; Schiapparelli et al., 2014) into nascent proteins. The bio‐orthogonal non‐canonical amino acid tagging (BONCAT) method employs click‐chemistry to covalently link the AHA‐labeled nascent proteins via the AHA's azide group to biotin‐alkyne probes for enrichment via streptavidin (Chang et al., 2010; Dieterich et al., 2006; Hodas et al., 2012; Howden et al., 2013; van Bergen et al., 2022) (see Figure 4d). Using BONCAT, translational changes at very early time points (e.g., 10 min labeling time) can be monitored by MS. However, its application requires methionine starvation during cell culture and adverse effects can occur due to copper toxicity during the click reaction (Chang et al., 2010).

To leverage the strengths of both chemical tagging and metabolic SIL, BONCAT has been combined with pSILAC, a method termed QuaNCAT (Bagert et al., 2014; Howden et al., 2013). In an analogous method, termed puromycin‐ and SILAC labeling‐based nascent polypeptidome profiling (pSNAP), puromycin labeling and pSILAC have been combined, followed by the enrichment of nascent polypeptide chains using anti‐puromycin antibodies before MS analysis (Uchiyama et al., 2022). In each case, the pSILAC component provides an internal standard, facilitating the accurate measurement and direct comparison of protein synthesis rates between conditions while avoiding technical variability during sample processing. Both methods have been successfully employed to globally monitor rapid translational responses to different treatments, with labeling times of 10 min (QuaNCAT) and 30 min (pSNAP).

#### 3.3.3 Thermal proteome profiling

In recent years, proteomics methodology to globally measure protein stability and folding states in situ has been developed, revealing changes in protein states such as conformation changes or complex assembly during stress or in disease models (Le Sueur et al., 2022; Mateus et al., 2020). The basis of these methods is the Cellular Thermal Shift Assay (CETSA) (Martinez Molina et al., 2013). CETSA is based on the biophysical principle that proteins are more resistant to thermal denaturation when they are stabilized by a ligand, a metabolite, or protein–protein interaction. Thermal proteome profiling (TPP) and thermal proximity co‐aggregation (TPCA) scale the CETSA principle to a proteome‐wide level using defined temperature gradients (e.g., 37–67°C) and LC–MS analysis (Savitski et al., 2014; Tan et al., 2018). The versatility of TPP stems from its ability to detect changes in protein states across diverse biological contexts, enabling to assess in vivo protein stability, small molecule targets and the “metabolome‐proteome” interface. As proteins within the same physical complex tend to have nearly identical melting profiles, TPCA allows monitoring dynamic changes in protein–protein interactions, such as protein complexes that form or dissolve at specific stages of the eukaryotic cell cycle (Tan et al., 2018) or during infection (Hashimoto et al., 2020).

To achieve the high‐throughput precision required for TPP and TPCA, proteins are globally profiled using multiplexing MS technology. After cells are exposed to a temperature gradient, the soluble fractions are collected, and each temperature point is then “barcoded” with a TMT tag to quantify a protein's solubility across the entire temperature gradient by multiplexed MS analysis. For each protein, a sigmoidal dose–response model is fitted to the acquired temperature‐dependent data, a key parameter of which is the melting point (Tm), that is, the temperature at which 50% of the protein is lost due to denaturation and aggregation. Based on the analysis of Tm shifts or comparison of the entire melting profile in control and stressed cells, information on alterations of protein functional states can be obtained on a whole‐proteome level.

An inherent limitation of TPP concerns intrinsically disordered proteins and highly stable membrane proteins typically showing no clear, sigmoidal transition from soluble to insoluble within the standard temperature range applied. This renders approximately 25%–30% of the proteome invisible to TPP analysis. Furthermore, TPP lacks peptide level resolution, and thus specific binding site or the exact domain undergoing a conformational shift cannot be inferred (Mateus et al., 2020).

## 4 APPLICATIONS TO MITOCHONDRIAL PROTEOSTASIS RESEARCH

In the following sections, we present how the quantitative, functional proteomics approaches discussed above were utilized to study cellular principles and processes of mitostasis. These techniques have had a tremendous impact on the research field as demonstrated by numerous recent studies on mitochondrial stress response networks and proteostasis mechanisms. We mainly, but not exclusively, focus on studies investigating responses to mitochondrial import stress. We grouped the studies discussed according to the main proteomics approaches employed and describe the key findings obtained from these analyses and their implications.

### 4.1 Globally monitoring cellular responses to mitochondrial import stress

As mitochondria are both physically and functionally closely interconnected with the larger subcellular network, understanding the consequences of impaired mitochondrial biogenesis and dysfunction requires a holistic view through analysis of the entire cellular proteome.

#### 4.1.1 Identifying stress response networks

In seminal work, Wrobel et al. (2015) investigated the global cellular response to impaired mitochondrial protein import using *S. cerevisiae* translocase mutants. To this end, they moved away from traditional approaches using isolated mitochondria but instead performed whole‐cell proteomics of import‐deficient versus control cells using a SILAC‐MS approach. This quantitative analysis of changes in the cellular proteome upon mitochondrial import stress induction led to the discovery of a specific cellular stress response elicited by the over‐accumulation of toxic mitochondrial precursor proteins in the cytosol, termed Unfolded Protein Response activated by mistargeting of proteins (UPRam). It consists of two arms: enhancing proteasomal capacity for the efficient clearance of mislocalized mitochondrial precursors and fast translation attenuation to alleviate the burden of toxic precursor over‐accumulation in the cytosol. Remarkably, the SILAC data revealed increased levels of the chaperone complex constituents Irc25 and Poc4, suggesting that elevating proteasome assembly is a direct mechanism for increasing precursor degradation capacity. Notably, UPRam operates independently of the mitochondrial unfolded protein response, UPRmt, which responds to protein misfolding within the mitochondria by regulating mitochondrial chaperone and protease abundance (Krämer et al., 2023).

Building on these findings, Boos et al. (2019) utilized engineered “clogger” proteins to interfere with mitochondrial precursor import through the TOM complex in *S. cerevisiae* cells. These inducible clogger constructs, b2Δ‐DHFR and b2‐DHFR, contain a matrix or an IMS targeting sequence fused to mouse dihydroxyfolate reductase (DHFR) that forms a stabilized tightly folded structure upon treatment with methotrexate (MTX). Following clogger induction, which led to rapid precursor accumulation, cellular responses were monitored using multiplexed quantitative proteomics and transcriptomics. This time‐resolved study confirmed a pronounced increase in the abundance of cytosolic chaperones of the Hsp70/90 families and proteasomal subunits, indicating that the observed transcriptional “waves” are directly translated into a rapidly activated proteostatic defense system. Integration of whole‐cell proteome and transcriptome data indicated that UPRam (Wrobel et al., 2015) and mitoCPR (Weidberg & Amon, 2018) are downstream of the Hsf1‐ and Rpn4‐mediated core transcriptional response to mitochondrial import stress.

#### 4.1.2 Dissecting mitochondrial crosstalk

While the studies discussed above emphasize global cellular responses to a dysfunctional mitochondrial import system, recent work reports an alternative targeting route of newly synthesized mitochondrial proteins in yeast. In this ER surface‐mediated protein targeting (ER‐SURF) pathway, mitochondrial precursor proteins are routed to mitochondria via the ER surface (Hansen et al., 2018; Koch et al., 2021). Herrmann and colleagues (Koch et al., 2024) dissected this pathway using quantitative proteomic techniques. They first analyzed the effects of the loss of mitochondria‐ER contact sites (MERCS), which may promote the transfer of proteins from the ER to mitochondria, on the mitochondrial proteome of yeast deficient in such contacts, revealing a significant decrease in mitochondrial matrix and IM proteins in the mutant cells. This demonstrates that MERCS are important for the biogenesis and/or stability of distinct mitochondrial proteins. To identify the clients of the pathway, the authors analyzed mitochondria and ER affinity‐purified from an organelle‐enriched fraction from wildtype (WT) versus cells deficient for MERCS. This analysis showed that inner membrane proteins and further proteins with hydrophobic stretches in their sequences are the preferred clients of this pathway. One could envision that under conditions of mitochondrial protein import stress, hydrophobic precursors are retained in the ER, preventing clogging of the import channel and proteotoxic aggregation in the cytosol.

Expanding on the definition of ER‐SURF clients, a recent study by Langer and colleagues (Kroczek et al., 2026) provided further evidence for the ER's role as a spatial buffer during mitochondrial “clogging” stress. To resolve the organellar distribution of proteins under stress, the authors utilized subcellular fractionation to separate mitochondrial and ER‐enriched membrane fractions from cells. Consistent with the ER‐SURF model, their SILAC‐MS analysis revealed that while the total cellular abundance of mitochondrial precursors remained relatively stable, there was a significant shift in their localization. This finding bridges the gap between the specialized targeting described by Koch et al. (2024) and the broader mitochondrial stress response, suggesting that the ER‐SURF pathway may act as a “fail‐safe” when the standard TOM‐dependent import route is compromised.

In a previous study, Peikert and colleagues defined the mitochondrial “importome” of the protozoan parasite *Trypanosoma brucei* (Peikert et al., 2017). The experimental approach combines inducible knockdown of ATOM40, the functional equivalent to Tom40, with quantitative analysis of gradient‐purified mitochondria from ATOM40‐depleted versus wildtype cells using SILAC‐MS. Interestingly, levels of virtually all mitochondrial proteins were decreased upon ATOM40 knockdown while there was no pronounced protein population with increased abundance. Following this new “importomics” approach, a systematic screen of all other components of the atypical TOM (ATOM) complex revealed that only depletion of ATOM69, the receptor with preference for more hydrophobic proteins, led to a specific response: the recruitment of non‐mitochondrial proteins, including proteasomal subunits, to the organelle (Dewar et al., 2022). Three of these proteins were shown to function in a QC pathway that results in the proteasomal degradation of non‐imported mitochondrial proteins. These proteins are TbUbL1, a protein with N‐terminal ubiquitin‐like domain and a nuclear localization sequence, an E3 ubiquitin ligase (TbE3HECT1), and a protein of unknown function (Tb927.9.7200). TbUbL1 was shown to be localized to the nucleus. In the absence of ATOM69, a fraction of TbUbL1 is released to the cytosol where it can bind to mislocalized aggregation‐prone mitochondrial proteins to deliver them to the proteasome for degradation, thereby preventing proteotoxic stress.

### 4.2 Mapping protein assemblies to reveal mitostasis pathways and QC factors

Application of quantitative AP‐MS and proximity proteomics strategies to study mitochondrial proteostasis has significantly advanced our current understanding of an intricate cellular protein QC network that safeguards both cytosolic and mitochondrial proteomes under basal and stress conditions.

#### 4.2.1 Revealing mitochondrial protein sequestration

Sequestration of misfolded or aggregation‐prone proteins is a common protein QC principle to protect cells and mitochondria from proteotoxic damage. Recently, so‐called “MitoStores,” cytosolic protein granules enriched in mitochondrial precursor proteins with N‐terminal targeting sequences, were reported to serve as transient storage sites for non‐imported precursors when the mitochondrial protein import machinery is overloaded (Krämer et al., 2023). Through whole‐cell proteomics, levels of the disaggregase Hsp42 and the small heat shock protein Hsp104 were found to be increased in Δ*rpn4* cells (i.e., cells deficient in the transcriptional upregulation of ubiquitin–proteasome system components) during mitochondrial import stress. Through qAP‐MS analysis, many nucleus‐encoded mitochondrial proteins with N‐terminal presequences were identified to co‐purify with Hsp104 during import stress, leading to the concept of MitoStores.

Within mitochondria, a specialized intramitochondrial protein QC compartment termed “IMiQ” has been identified and described as stress‐induced protein aggregates that are shielded by a shell of Hsp70 proteins that isolates the proteotoxic polypeptides (Ruland et al., 2025). The finding is based experimentally on qAP‐MS utilizing an artificial, aggregation‐prone construct targeted to mitochondria, which revealed that IMiQ provides an intramitochondrial sink for respiratory chain components under protein aggregation conditions.

A complementary intramitochondrial sequestration pathway termed “MitoTrap” (mitochondrial triage of precursor proteins) was identified using APEX2 specifically targeted to the IMS or matrix for PL expressed in WT cells and cells lacking the F_0_F_1_‐ATPase subunit Atp6. *ATP6* deletion results in a decreased inner membrane potential (Flohr et al., 2025). The cells are still import‐competent, but at reduced rates. Quantitative MS analysis of streptavidin‐enriched biotinylated proteins revealed that distinct proteins—primarily mitochondrial ribosomal proteins lacking canonical N‐terminal targeting sequences—are sequestered in the IMS of the energetically compromised mitochondria. Based on qAP‐MS analysis, the authors showed that trapping Mrp17 (bS6m) in the IMS of compromised mitochondria prevents its mislocalization to the nucleus where it otherwise interfered with the assembly machinery of cytosolic ribosomes. Collectively, the interactome data characterized MitoTrap as a QC system that prevents mistargeting of mitoribosomal proteins to the nucleus to maintain proper assembly of cytosolic ribosomes when mitochondrial protein import is affected.

In addition to intramitochondrial protein quality control compartment (IMIQ) and MitoTrap, qAP‐MS further identified different types of spatially defined stress‐induced protein aggregates in yeast mitochondria: more transient Hsp78‐containing aggregates and more stable Var1 bodies (Bertgen et al., 2024). Hsp78‐GFP complexes purified from *rho0* cells, which lack the mitochondrial genome and, thus, cannot assemble mitoribosomes, were enriched in many mitochondrial proteins including ribosomal proteins. This Hsp78‐dependent sequestration of proteins into transient, reversible aggregates prevents their degradation by the Lon protease Pim1. Var1 (uS3M) is a mitochondrial‐encoded protein of the small subunit of the mitochondrial ribosome. A plasmid‐encoded variant of this protein, fused to a mitochondrial targeting sequence, was used to purify Var1 complexes from mitochondrial fractions and to analyze their composition by qAP‐MS. Since Var1 bodies were highly enriched in proteins of the mitochondrial proteostasis network including chaperones (except for Hsp78) and proteases, it was suggested that Var1 bodies sequester misfolded matrix proteins to protect the mitochondria from proteotoxic stress. Consistent with reversible sequestration, an independent qAP‐MS study demonstrated that Hsp78 promotes disaggregation of protein aggregates following heat stress relief, thereby restoring essential mitochondrial functions such as tricarboxylic acid cycle (TCA) cycle activity, oxidative phosphorylation (OXPHOS), protein import and synthesis (Jaworek et al., 2022).

Furthermore, APEX2‐mediated proximity proteomics and qAP‐MS revealed an enrichment of aggregation‐prone mitochondrial inner membrane and mitoribosomal precursor proteins in SQSTM1/p62 aggregates of aneuploid cells (Amponsah et al., 2025). These cells exhibit an imbalance in protein expression and folding due to increased numbers of individual chromosomes. Sequestration of these mitochondrial precursor proteins in p62 bodies protects the cells from toxic protein aggregation. However, the authors also report impaired mitochondrial function, including elevated reactive oxygen species (ROS) levels, reduced mitochondrial DNA abundance, and decreased protein import. These defects may, at least in part, result from the sequestration of mitochondrial precursor proteins in p62 bodies, thereby compromising the mitochondrial protein import machinery and reducing overall import capacity.

#### 4.2.2 Identifying clearance and degradation mechanisms

Apart from sequestration and QC pathways at the outer mitochondrial membrane (OM), MS‐based interactome studies have revealed complementary pathways that preserve proteome integrity through cytosolic clearance or selective mitochondrial degradation. In HEK293T cells with impaired mitochondrial protein import, evoked by mitochondrial Complex I deficiency, levels of the chaperone HSPB1 and PSMB9‐containing immunoproteasomes are increased. This was revealed by quantitative MS analysis of cytosolic soluble fractions and protein aggregates from *NDUFA11* knockout cells and affinity‐purified proteosomes from *NDUFA11* or *NDUFA13* knockout versus control cells (Kim et al., 2023). Induction of the immunoproteasome under mitochondrial import stress conditions enhances the cytosolic clearance capacity for non‐imported precursor proteins, thereby protecting the cell against proteotoxicity.

Following a multifaceted proteomics strategy that included the identification of proteins associated with a proteolytically inactive variant of AFG3L2 (a catalytic subunit of the human m‐AAA protease with key role in IM proteostasis) using qAP‐MS as well as changes in the turnover rates of mitochondrial proteins in cells lacking the AFG3L2 interaction partner TMBIM5 using dynamic SILAC, Langer and colleagues revealed the following regulatory mechanism of mitochondrial proteostasis: Under normal conditions, TMBIM5 inhibits the protease activity of AFG3L2. In case of persistent hyperpolarization of the IM, however, TMBIM5 is degraded, which leads to activation of AFG3L2 and, in the following, to remodeling of the mitochondrial proteome to reduce production of ROS. The TMBIM5‐AFG3L2 system thereby links mitostasis to the energetic state of the organelle (Patron et al., 2022).

#### 4.2.3 Discovering quality control mechanisms at the TOM complex

Newly synthesized mitochondrial precursor proteins enter mitochondria through the TOM complex, and previous work showed that stalled precursors elicit cellular stress responses and specific mechanisms to remove non‐imported precursors in the cytosol (Boos et al., 2019; Mårtensson et al., 2019; Dewar et al., 2022; Pfanner et al., 2025; Schulte et al., 2023; Wang & Chen, 2015; Weidberg & Amon, 2018; Wrobel et al., 2015). To safeguard mitochondrial protein import, different protein QC mechanisms operate at the TOM complex, which thus represents a central QC checkpoint at the cytosol‐mitochondria interface (Backes et al., 2021; Mårtensson et al., 2019; McMinimy et al., 2024; Opaliński et al., 2018; Schulte et al., 2023). Detailed characterization of TOM assemblies using MS‐based proteomics technologies has paved the way for making these recent discoveries.

Mapping the interactome of Tom22 by SILAC‐AP‐MS in yeast revealed a specific association with the cytosolic J‐protein Xdj1 (Opaliński et al., 2018), whereas Tom70 functionally partners with the J‐protein Djp1. By binding to hydrophobic precursor proteins, Xdj1 and Djp1 keep them in a soluble, import‐competent state, thereby preventing aggregation and clogging of the translocase. Consistent with this stabilizing role, an independent study employing whole‐cell proteomics of WT versus Δ*tom70/71* cells to identify Tom70/71 clients and qAP‐MS of an unrelated chaperone‐binding protein artificially tethered to the OM established Tom70 as a chaperone‐tethering hub in yeast and defined the recruitment of chaperones to the OM to protect the cytosol from toxic aggregation of mitochondrial precursors—and not its role as import receptor—as a key function for Tom70 in vivo (Backes et al., 2021). Moreover, the Tom22 interactome includes Ubx2 (Opaliński et al., 2018), which recruits the AAA ATPase Cdc48 (p97/VCP in mammals) to the OM (Mårtensson et al., 2019). The Ubx2‐Cdc48 module functions within the constitutive QC pathway mitoTAD (mitochondrial protein translocation‐associated degradation), which continuously monitors the TOM complex and extracts stalled precursors under non‐stress conditions.

Quantitative proteome and interactome studies further led to discovery of a mechanism that protects the cell from reductive stress and acts at the TOM complex (McMinimy et al., 2024). Although ROS are generally known to have damaging effects, cells require a basal level of ROS to maintain homeostasis, and persistent ROS depletion induces a reactive stress response (Manford et al., 2020). Rapé and colleagues discovered that the central enzyme of this stress response pathway, the E3 ligase Cullin‐2‐FEM homolog B (CUL2^FEM1B^), is recruited to the TOM complex via FEM1B‐TOM20 interaction, where it senses Complex III‐derived ROS. By degradation of CUL2^FEM1B^ substrates under low, but not high ROS levels at the TOM complex, the E3 ligase modulates import of mitochondrial precursors in dependency of the prevailing condition. In case of high ROS levels, CUL2^FEM1B^ substrates are stabilized at the TOM complex, thereby reducing mitochondrial protein import.

Complementary to interactome studies of TOM components, CP provides a holistic view on the mitochondrial organizing network. Refinement of CP by cryo‐slicing BN‐MS allowed to establish a high‐resolution complexome of yeast mitochondria, referred to as MitCOM (Schulte et al., 2023). This analysis was based on abundance‐mass profiles of >1800 proteins (>90% of MitoCoP) (Morgenstern et al., 2021) and enabled the identification of several transient QC factors operating in the vicinity of the TOM40 import channel. Among these proteins are the cytosolic E3 ubiquitin ligase Rsp5, which in the study was shown to mediate ubiquitylation of mitochondrial precursor proteins at the TOM complex, and the OM‐localized deubiquitylase Ubp16, a homolog of mammalian USP30 (Nakamura & Hirose, 2008), which counteracts protein ubiquitylation by removing ubiquitin chains. Another TOM40‐associated factor identified in this study is the peptidyl‐tRNA hydrolase Pth2. Together with Dsk2, a yeast homolog of mammalian ubiquilins, Pth2 functions in a constitutive QC pathway that facilitates the clearance of non‐imported precursor proteins by targeting them to the proteasome for degradation, thus defining a surveillance mechanism that operates independently of the previously identified mitoTAD pathway. Together, these findings show that mitochondrial protein import is highly safeguarded by coordinated ubiquitylation, deubiquitylation, and degradation mechanisms acting at the TOM complex. In addition, the complexome revealed that a pool of the OM protein Om14 is directly associated with the TOM complex. Since Om14 acts as a receptor for cytosolic ribosomes, its interaction with the import machinery suggests a direct link between cytosolic translation and mitochondrial protein import. Such an interface could represent a checkpoint for the modulation of mitochondrial protein biogenesis upon mitochondrial stress.

CP also provided a comprehensive view onto the molecular organization of the human MitoCoP with an analysis depth of 116 BN‐gel slices in two independent replicates showing a high correlation (Morgenstern et al., 2021). Based on co‐migration profiles and hierarchical clustering, the association of newly identified MitoCoP proteins with central machineries of the mitochondrial organizing network was revealed. This includes C3orf33, a so far unknown member of the prohibitin‐stromatin family, in connection with the prohibitin complex, and TMEM256 as new interactor of the TIM23 translocase. In addition to the binding to TIM23, TMEM256 was found to associate with OXPHOS complexes and the prohibitins. Since TMEM256‐silenced cells showed a moderate import defect, it may function in regulating TIM23 stability via the prohibitins (Elancheliyan et al., 2024). Exploration of the human MitoCoP complexome is expected to reveal additional, so far unknown factors involved in mitostasis.

### 4.3 Interrogating proteome dynamics to uncover fast regulation

Recent dynamic proteomics methods have emerged as new powerful approaches for gaining unprecedented insights into mitostasis processes based on quantitative data on protein half‐lives, protein synthesis, and conformational changes on a proteome‐wide level. The kinetic data obtained by different approaches suggest that maintenance of the mitochondrial organizing protein network and proteostasis responses to mitochondrial stress are governed by highly heterogeneous protein dynamics rather than uniform kinetics.

#### 4.3.1 Determining immediate early translational changes to mitochondrial membrane stress

Mitochondrial stress leads to fast attenuation of cap‐dependent translation along with the induction of cap‐independent selective translation of cytoprotective factors via the ISR (Ryoo & Vasudevan, 2017; Sasaki et al., 2020). To monitor fast translational reprogramming during early stages of mitochondrial stress induced by inner membrane depolarization using carbonyl cyanide m‐chlorophenyl hydrazone (CCCP) (0–1 h), Stępkowski and colleagues (Stępkowski et al., 2024) performed nascent proteomics using BONCAT. They found widespread changes in the nascent proteome, with about the same number of nascent proteins decreased or increased in abundance after 1 h of CCCP treatment, including the upregulation of the transcriptional regulators ATF4 and NRF2. Their kinetic analysis showed that cytosolic and mitochondrial translation recover with distinct temporal profiles, generating a transient desynchronization between the synthesis of nucleus‐encoded and mitochondria‐encoded proteins, with a faster resynthesis of constituents of mitochondrial ribosomes compared to cytosolic ribosomes during recovery. Importantly, these shifts occur independently of steady‐state protein abundance levels, indicating direct translational control rather than secondary effects of protein turnover. Mechanistically, modulation of elongation dynamics, including reduced abundance of the eukaryotic elongation factor EEF1A1, was sufficient to recapitulate stress‐induced proteome remodeling (Stępkowski et al., 2024).

Of note, this translational buffering aligns with earlier mechanistic studies demonstrating that mitochondrial import stress elicits a coordinated cytosolic proteostasis response to prevent toxic precursor accumulation (Schäfer et al., 2022). In this work, the mePROD method has been adapted for use in isolated mitochondria to achieve high‐resolution mapping of mitochondria‐specific translation and protein import dynamics. This allows for the detection of subtle changes in the translocation of nucleus‐encoded proteins into the mitochondria under stress, providing a detailed kinetic view of how mitochondrial import failure or translocase “clogging” immediately reshapes the organellar proteome.

Together, these findings establish that mitochondrial stress triggers an upstream translational buffering mechanism that limits precursor overload, thereby safeguarding mitochondrial import capacity and reinforcing mitostasis.

#### 4.3.2 Assessing changes in protein stability under import stress

To monitor changes in thermal stability and abundance of newly synthesized and mature proteins in situ upon clogging of the TOM complex (Groh et al., 2023) implemented a combined pulsed SILAC‐2D‐TPP (Becher et al., 2018) approach. The analysis revealed that blocking of the mitochondrial precursor pathway rapidly imprints a characteristic signature on the thermal stability of the cellular proteome, even before total protein levels were affected. The analysis revealed that blocking of the mitochondrial precursor pathway rapidly imprints a characteristic signature on the thermal stability of the cellular proteome, even before total protein levels were affected. This was highlighted by a rapid decrease in the thermal stability of cytosolic chaperones, specifically the Hsp70 proteins Ssa1 and Ssa2, and the mitochondrial chaperonin Hsp60, suggesting a “sequestration effect” where hydrophobic non‐imported mitochondrial precursors accumulate in the cytosol and titrate away these key molecular chaperones, eventually leading to a global collapse of cellular folding capacity (Krämer et al., 2023). Interestingly, many ribosomal proteins positioned at the exit tunnel of the cytosolic translation machinery exhibited a marked increase in thermal stability, implying a structure–function relation underlying the fast attenuation of cytosolic translation during mitochondrial import stress (Boos et al., 2019; Wrobel et al., 2015).

Further insight into the intrinsic stability landscape of mitochondrial proteins is provided by a large cross‐species “meltome atlas” (Jarzab et al., 2020). This comprehensive dataset shows that mitochondrial proteins form a distinct thermodynamic class within a cellular proteome. Subunits of the same mitochondrial complex typically display strongly correlated melting behavior, demonstrating that TPP captures organellar protein–protein interactions in situ. Core components of the OXPHOS machinery, including respiratory chain subunits and ATP‐synthase complexes, ranked among the most thermally stable proteins detected, consistent with their dense membrane embedding, extensive cofactor binding, and highly cooperative multi‐subunit assembly. In line with this, respiration was maintained up to 46°C in human mitochondria. Conversely, mitochondrial proteins involved in RNA processing, translation, and metabolic pathways within the mitochondrial matrix exhibited comparatively lower and more heterogeneous thermal stability, reflecting dynamic association and regulatory flexibility.

#### 4.3.3 Uncovering regulation points within the mitochondrial organizing network

Mitochondrial protein turnover is not a uniform process but a complex mosaic of varied degradation rates. While previous whole‐cell studies (Fornasiero et al., 2018; Mathieson et al., 2018; Zecha et al., 2018) provided foundational turnover data, they covered only a limited fraction of the mitochondrial proteome and reported average turnover values for mature and precursor forms, as well as for multi‐localized mitochondrial proteins. Dynamic SILAC combined with mitochondria purification showed that OXPHOS complexes exhibit vastly different production‐to‐assembly ratios (Bogenhagen & Haley, 2020). While the assembly of Complex V is highly efficient with minimal excess subunit synthesis, pronounced oversynthesis of numerous Complex I proteins, particularly those within the matrix‐exposed N and Q domains, was observed. These unassembled “orphan” subunits do not persist and are typically degraded within 3 h, contributing to the short half‐lives observed for these subunits in global turnover studies (Fornasiero et al., 2018; Zecha et al., 2018).

By following an advanced mitochondria‐specific dynamic SILAC‐MS approach using Hela and Huh7 cells, the MitoCoP project established the most comprehensive protein‐half‐life map of the human mitochondrial organizing network (Morgenstern et al., 2021). Mitochondria‐specific half‐lives of over 830 mitochondrial proteins were determined, revealing a remarkable kinetic range spanning three orders of magnitude from 1.6 h to several months. Furthermore, mitochondrial protein half‐lives vary significantly within the same multi‐subunit complex or pathway, a characteristic that is not limited to OXPHOS complexes. This half‐life heterogeneity is not random but suggests a sophisticated regulatory architecture. Within the translocase network, the membrane‐embedded core subunits of the TOM complex (TOMM40 and TOMM22) and the sorting and assembly machinery (SAM) complex (SAMM50) are extremely long‐lived, providing a stable structural framework for protein import across and into the mitochondrial OM. Conversely, the presequence receptor of the TOM complex, TOMM20, is very short‐lived. Since TOMM20 initially recognizes import‐competent precursor proteins, its fast turnover may likely provide a direct mechanism for modulating precursor import during stress, but also to rapidly replace the cytosol‐facing receptor after damage. Remarkably different half‐lives were also observed for different subunit isoforms of the TIM23 machinery facilitating precursor protein import into the mitochondrial IM and matrix. These include the TIM23 core subunits TIMM17A/TIMM17B and the PAM subunits GrpEL1/GrpEL2 and DNAJC15/DNAJC19. The identification of TIMM17B as long‐lived and TIMM17A as short‐lived isoform is in line with previous reports: while TIMM17A forms a “housekeeping” variant of the TIM23 translocase, TIMM17A was shown to be rapidly degraded in response to stress (Maruszczak et al., 2024; Opalińska et al., 2018; Rainbolt et al., 2013). Collectively, a picture emerges where the coexistence of short‐ and long‐lived TIM23 complexes provides a direct mechanism for adjusting mitochondrial precursor import capacity by rapid decay of short‐lived TIM23 subunits.

## 5 CONCLUDING REMARKS

By providing technology for systematic and accurate protein identification and quantification, MS‐based proteomics has evolved into a cornerstone method for the exploration of mitostasis. Whereas traditional analysis focused on isolated mitochondria and then cell lysates, the field is increasingly aware of the importance of placing the organelle into a cellular context to better understand extra‐mitochondrial QC and proteostasis networks, and to uncover organelle crosstalk. Importantly, a large portion of the cellular proteome is multi‐localized, and dynamic change in subcellular protein localization emerges as a driving force for human health and disease (Sigaeva et al., 2026). Quantitative MS technology is most appropriate for the detailed spatiotemporal analysis of the proteome employing PL and subcellular fractionation methods (Christopher et al., 2021). Here, use of DIA‐MS with narrow window precursor isolation (Guzman et al., 2024) offers even deeper coverage, higher sensitivity and faster throughput, providing a powerful proteome‐wide readout that can be easily reanalyzed in the light of new findings and different functional hypotheses (Kitata et al., 2023). Moreover, MS instrumentation implementing concepts of gas‐phase ion trapping for increased duty cycle enables the analysis of a growing number of replicates in reasonable time (Guzman et al., 2026; Skowronek et al., 2025), thereby pushing not only boundaries in sample throughput but also increasing statistical power of the results even for small effect sizes.

A further important frontier of mitostasis research lies in the deciphering of regulatory signals mediated by post‐translational modifications. For example, phosphorylation plays a central role in adapting mitochondrial function to different tissues (Hansen et al., 2024) or metabolic states (Grimsrud et al., 2012) and is tightly regulated (Niemi et al., 2023). Similar protein control through ubiquitination and proteasomal degradation plays a central role for mitostasis (Antico et al., 2021). In this direction, a largely uncharted field lies in the systematic unbiased analysis of signaling pathways underlying the immediate activation and regulation of cellular proteostasis networks under acute mitochondrial stress. For this, advanced phosphoproteomics and ubiquitinomics methodologies are already at hand (Faisst et al., 2026; Hansen et al., 2021; Oliinyk et al., 2024). Integrating signaling dynamics with protein turnover, ideally in a temporal and spatially resolved manner, will provide a more nuanced view of how cells maintain mitochondrial function and integrity, eventually offering new therapeutic avenues to treat diseases linked to mitochondrial dysfunction.

## AUTHOR CONTRIBUTIONS

**Hirak Das:** Writing – original draft. **Silke Oeljeklaus:** Writing – original draft; writing – review and editing; supervision; visualization. **Sristi Chakroborty:** Writing – original draft; visualization; writing – review and editing. **Julian Bender:** Writing – original draft; visualization; writing – review and editing. **Lakshita Sharma:** Writing – original draft; visualization; writing – review and editing. **Bettina Warscheid:** Conceptualization; funding acquisition; writing – original draft; writing – review and editing; supervision.

## FUNDING INFORMATION

Research in the lab of Bettina Warscheid was supported by the Deutsche Forschungsgemeinschaft (DFG, German Research Foundation) through grants GRK2243/2 (project number 285767414) and SPP2453 (project number 541758684; WA 1598/7‐1).

## CONFLICT OF INTEREST STATEMENT

The authors declare that there are no conflicts of interest.

## Data Availability

Data sharing not applicable to this article as no datasets were generated or analyzed during the current study.
